# Diversity and Activity of Bacteria Cultured from a Cup—The Sponge *Calyx nicaeensis*

**DOI:** 10.3390/md22100440

**Published:** 2024-09-26

**Authors:** Lynne Itelson, Mayan Merav, Shai Haymi, Shmuel Carmeli, Micha Ilan

**Affiliations:** 1 School of Zoology, Faculty of Life Science, Tel Aviv University, Tel Aviv 6997801, Israel; shai12132@gmail.com; 2 School of Chemistry, Raymond and Beverly Sackler Faculty of Exact Sciences, Tel Aviv University, Tel Aviv 6997801, Israel; mayanmv@gmail.com (M.M.); carmeli@tauex.tau.ac.il (S.C.)

**Keywords:** sponges, mesophotic, *Calyx nicaeensis*, antibiotics, *Paenisporosarcina indica*, biosurfactants

## Abstract

Marine sponges are well-known for hosting rich microbial communities. Sponges are the most prolific source of marine bioactive compounds, which are frequently synthesized by their associated microbiota. *Calyx nicaeensis* is an endemic Mediterranean sponge with scarce information regarding its (bioactive) secondary metabolites. East Mediterranean specimens of mesophotic *C. nicaeensis* have never been studied. Moreover, no research has inspected its associated bacteria. Thus, we studied the sponge’s bacterial diversity and examined bacterial interspecific interactions in search of a promising antibacterial candidate. Such novel antimicrobial agents are needed since extensive antibiotic use leads to bacterial drug resistance. Bacteria cultivation yielded 90 operational taxonomic units (OTUs). A competition assay enabled the testing of interspecific interactions between the cultured OTUs. The highest-ranked antagonistic bacterium, identified as *Paenisporosarcina indica* (previously never found in marine or cold habitats), was mass cultured, extracted, and separated using size exclusion and reversed-phase chromatographic methods, guided by antibacterial activity. A pure compound was isolated and identified as 3-oxy-anteiso-C_15_-fatty acid-lichenysin. Five additional active compounds await final cleaning; however, they are lichenysins and surfactins. These are the first antibacterial compounds identified from either the *C. nicaeensis* sponge or *P. indica* bacterium. It also revealed that the genus *Bacillus* is not an exclusive producer of lichenysin and surfactin.

## 1. Introduction

Sponges (Phylum Porifera) are simple multi-cellular sessile organisms and are considered the oldest metazoans living on earth [1,2]. They are highly versatile and can thrive in a variety of habitats [3,4,5]. They are considered highly efficient filter feeders that can filter up to 24,000 L per 1 kg (wet weight) in a single day [6,7]. Sponges rely on a complex network of water-pipes and filtration chambers for nutrition and respiration. They host rich microbial communities from all three domains of life (Bacteria, Archaea, and Eukarya) that can comprise up to 40% of the sponge’s volume [8,9]. In this relationship, the sponge provides the habitat and nutrition for these microorganisms, while in return, the microorganisms may provide important metabolic functions that support the host’s survival by contributing to its nutrition, development, immunity, and defense [10,11]. The efficient filter-feeding strategy exposes sponges to a large volume of sea water, wherein a diverse array of bacteria is found, with concentrations ranging from ~10^5^ to 10^6^ bacteria per mL of seawater [12,13,14]. With this concentration and such filtration rates, it is estimated that 2.4 × 10^13^ bacterial cells pass into the sponge each day [15]. Thus, even if a particular bacterium species is present at just 1 cell/mL, the sponge could filter 24 million cells of this bacterium from the water column in a single day [8,15]. Sponges retain unique bacterial communities that differ from those present in the surrounding water, which indicates a specific symbiotic relationship with the sponge host. The microbial growth within the sponge may be regulated by the host sponge (using specific growth inhibitors, through expulsion or phagocytosis of the sponge-associated bacteria) or by microbial interspecific interactions [16,17]. The complex sponge–symbiont interactions are not fully understood; thus, mimicking them under artificial growth conditions in the lab remains challenging but also promising to enable the growth of the thus far unculturable microorganisms [18,19,20,21]. The genetic marker 16S ribosomal RNA (rRNA) revealed the extensive diversity and host-specific characteristics of sponge-associated bacteria [22]. More than 60 bacterial phyla lack cultured representatives, with cultivation values of associated bacteria estimated at 0.1–14% [10,23,24,25,26,27]. Given the generally low levels of bacterial cultivation success, there remains a vast array of unexamined bioactive compounds potentially produced by sponge-associated bacteria, offering possible pharmaceutical and biotechnological applications. In general, bioactive compounds from the marine environment are naturally produced by various marine organisms, such as algae, bacteria, fungi, sponges, corals, etc. Sponges are well-known for their ability to produce an enormous array of natural compounds [28]. To date, over 2659 compounds derived from sponges have been identified, with many exhibiting distinctive qualities. [29]. These natural compounds show a wide range of bioactivities, such as anticancer, antiviral, and antimicrobial bioactivities [30]. These secondary metabolites can be produced by either the sponge-host cells or by its associated microbiota [31]. To obtain a large quantity of compounds from sponge extraction, a substantial mass of sponge is required. Developing marine natural products using attractive lead compounds requires the application of different production methods that do not rely on harvesting sponges. One solution to the supply problem is the cultivation of host-associated bacteria that produce the desired compound. Host-associated bacteria cultivation has many advantages over sponge harvesting. For example, cultivation is more sustainable and offers a potentially unlimited supply of metabolites at a low cost through fermentation [8]. The host often holds minute quantities of secondary metabolites, and by using culturable bacteria, collecting and affecting natural sponge populations could be prevented [32,33]. Finally, cultured bacteria are amenable to genetic manipulation options that could improve natural product production and efficacy [34].

**Figure 1:**
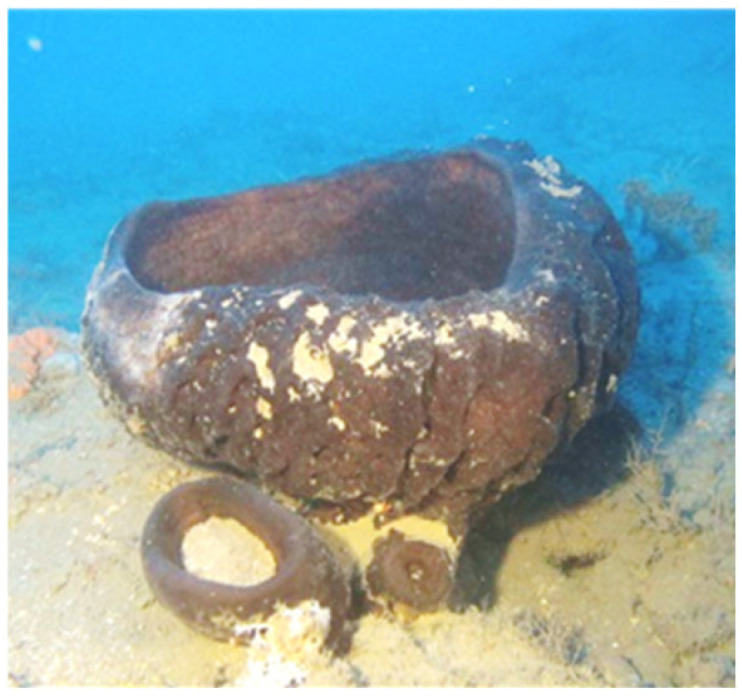
*Calyx nicaeensis* sponge (in situ at 99 m). Photo by Micha Ilan’s Lab.

In recent years there has been a growing demand for new sources of antibiotics due to the increasing prevalence of drug-resistant bacteria. Sponge-associated bacteria that are confined within their host compete for resources, hence exhibiting antagonistic antibacterial activity [35], and were indeed found to produce antibacterial active compounds. This led to an increased interest in sponge-associated bacteria as a source of new antibiotic compounds [36,37]. Approximately 800 antibiotic compounds have been isolated from marine sponges [35,38,39]. Shallow-water sponges (<30 m) are located at depths accessible to humans, have been widely studied over the past few decades, and are also known for their potential biotechnological applications, while deeper habitats are much less known due to challenging and costly logistics [40,41,42]. The underexplored mesophotic habitats hold a different set of traits than the shallow water, such as reduced light intensity, lower temperature fluctuations, lower human impact, and environment-related disturbances. Therefore, the current study focused on a mesophotic sponge to increase the potential to find new antibacterial compounds. The sponge, *Calyx nicaeensis* (Porifera, Demospongiae, Haplosclerida, Phloeodictyidae), is a cup-shaped species (Figure 1) that is endemic to the Mediterranean Sea. This species is found in a wide range of habitats and depths [43] and is commonly observed at depths between 5 and 55 m but also reaches down to 400 m [44]. Despite its rare distribution in the Mediterranean [43], along the Israeli coast, this sponge is more common in the mesophotic depths (100–120 m) [45]. To the best of our knowledge, no work has been carried out on the microbial interspecific interactions within this species. Moreover, little information is known about the *C. nicaeensis* microbiome and its secondary metabolites. Unique steroidal compounds have been isolated from *C. nicaeensis* over the years [46,47,48,49], including cyclopropane-containing sterols named calysterols [50,51,52]. Dogan et al. (2018) [53] reported that *C. nicaeensis* extracts have antimicrobial traits but did not identify the specific active compound. Therefore, the mesophotic sponge *C. nicaeensis* was chosen as a candidate sponge to screen its associated bacterial community.

## 2. Results

### 2.1. Microbial Diversity of C. nicaeensis—Associated Cultivable Bacteria

**Figure 2:**
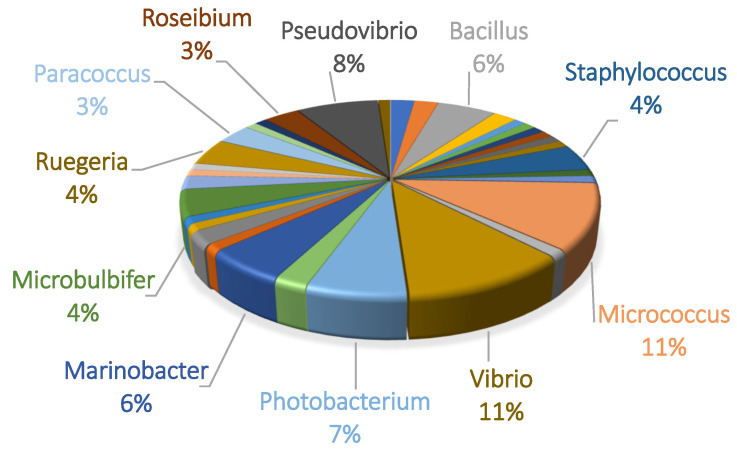
Distribution of the sponge-associated OTUs cultivated from *C. nicaeensis* per genus. Genera above 3% are named in this figure.

A total of 319 isolates with different morphology traits were obtained from two *C. nicaeensis* specimens and cultured on four different media. The 16S rRNA gene was successfully sequenced for only 258 of these isolates. The sequenced isolates were clustered into 90 OTUs. The OTUs were classified to their closest phylogenetic neighbors, resulting in three phyla, four classes, 11 orders, 17 families, and 34 genera. The phylogenetic relations between OTUs with bootstrap values using partial 16S rDNA sequences are presented in Figure S1. Proteobacteria was the dominant cultivated phylum, accounting for 62% of the OTUs, followed by Firmicutes 26%, and Actinobacteria 12%. Within Proteobacteria, 64.3% of representatives were from the class Gammaproteobacteria, and 35.7% were from Alphaproteobacteria. The dominant cultivable genera were *Micrococcus* and *Vibrio*, each counting for 11% (Figure 2). OTU #54 had less than 97% similarity to any validly described species; thus, it could represent a new cultured bacterial species. Out of all the tested media, the ISP2 medium had the highest OTU richness, with 46.5% of the total OTUs being observed growing on it. The second highest OTU richness was observed on the MA medium, followed by LB and SCA, respectively (39.8%, 32.1%, and 15.5%). ISP2 and MA shared the highest number of OTUs among all growth media pairs at 4.4%. ISP2, LB, and MA had the greatest overlap of the three groups at 4.4%. All media shared 3.3% of the OTUs. Most of the OTUs (78%) were specific to one type of medium, 13% grew on two media, and 4.5% grew on three and four media. The addition of antibiotics did not enhance Gram-positive over Gram-negative bacteria growth percentages; however, little overlap was seen between OTUs grown on plates with and without antibiotics. Of the total OTUs, 32.2% grew with antibiotics, 44.4% grew without antibiotics, and 23.3% grew under both conditions.

### 2.2. Competition Assay—Antibacterial Screening

**Figure 3:**
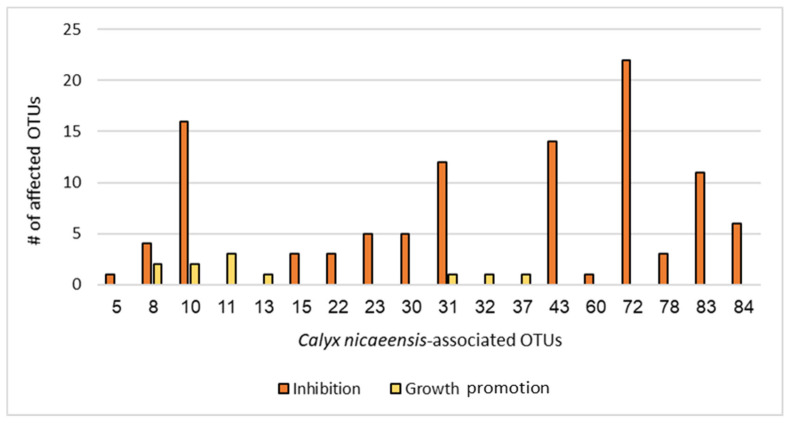
Growth inhibition and promotion effects of 18 *C. nicaeensis*-associated OTUs tested in the competition assay. (_)—inhibition, (_)—promotion.

**Figure 4:**
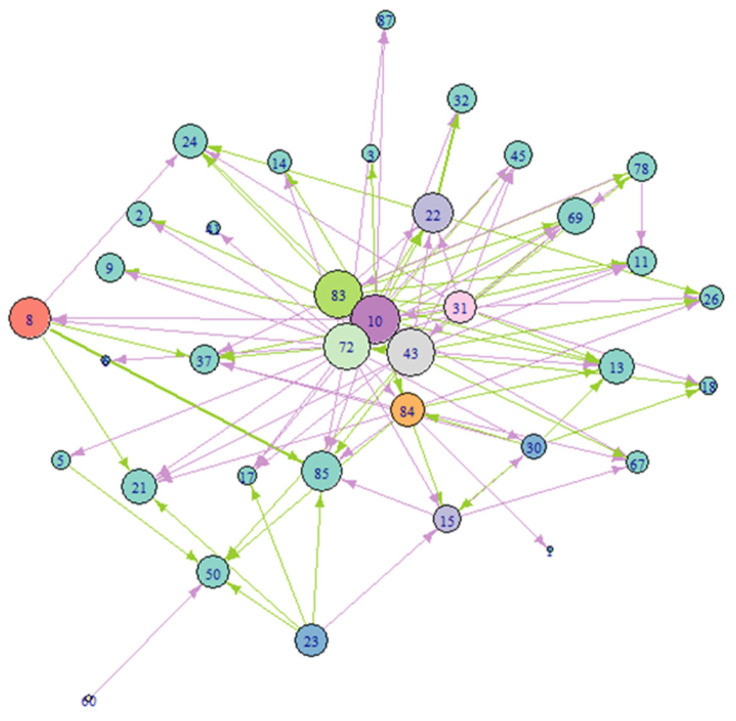
Interspecific interactions assessed using eigenvector centrality between the cultivated OTUs from the *C. nicaeensis* sponge. Numbers represent different OTUs. Larger nodes represent the eigenvector centrality score. Different colors mark bacteria that inhibit a large number of bacteria. Green arrows represent inhibition >1 cm and purple >1 cm. The four OTUs with the strongest inhibitions were 83, 72, 43, and 10.

The aim of the competition assay was to test the interspecific interactions between the cultivated *C. nicaeensis*-associated bacteria and to better understand the sponge as a holobiont. Additionally, the assay sought to identify bacteria with the biotechnological potential of antibacterial properties. A screening test was performed for ISP2 isolates (22 Gram-positive OTUs) against 40 Gram-positive and -negative OTUs isolated from *C. nicaeensis*. Antibacterial activity against at least 1 of these 40 target bacteria was found in 19 out of the 22 OTUs isolates (73%), belonging to two phyla and six genera. Most active isolates were affiliated with the phylum Firmicutes (64%), followed by Actinobacteria (36%). The active isolates were chosen to continue testing in triplicates test (90 did not have results due to contamination, leaving 18 OTUs to be tested). From the triplicate results, it was found that OTU #72, which belongs to the genus *Paenisporosarcina*, inhibited 22 test bacteria—the highest number of inhibited bacteria by a single OTU. About half (49% 52/106) exhibited strong inhibition streaks (>1 cm). Generally, the sponge-associated bacteria did not demonstrate a consistent tendency toward either strong or weak inhibition. However, we can see a pattern of strong or weak inhibition when looking at each bacterium on its own. For example, while OTUs #22 and 23 strongly inhibited a small number of other OTUs (three and five, respectively), OTU #72 weakly inhibited the maximum number of OTUs (22). On the other hand, OTU #10 strongly inhibited a large number of OTUs (16). Bacteria growth promotion mostly (7/11) had a strong effect. In addition, growth inhibition affected a large variety of bacteria. For example, OTU #72 inhibited OTUs from 15 different families. This is seen in most of the inhibiting OTUs. Growth promotion, on the other hand, had a narrower variety and was seen in only six OTUs belonging to three families. The maximum number of promoted OTUs by a single OTU was three (by OTU #11). Most of the tested bacteria either inhibited or promoted (15/18), while 3/18 did both (Figure 3). Interspecific interactions between tested OTUs were measured by using eigenvector centrality (Figure 4). Four bacteria showed the strongest scores, and their closest related genera are *Priesta* (#83), *Bacillus* (#43, #10), and *Paenisporosarcina* (#72). 

### 2.3. Chemical Extraction and Separations

Since OTU #72 was found to be the “strongest” competitor, it was mass-cultured and extracted. An initial experiment showed activity of the OTU #72 crude extracts from both medium and cell mass (in CH_2_Cl_2_/MeOH 1:1). The first mass production (13.8 L of medium) yielded 11.1 g of crude extract, while the second extraction resulted in 11.64 g (from MeOH) and 3.86 g (from CH_2_Cl_2_-DCM). In the second extraction, the bacteria cell mass extract wet weight was 28.5 g, yielding 3.42 g of extract (DCM and MeOH combined). This crude extract was tested against all bacteria isolates previously inhibited by OTU #72 in the competition assay. It was active against four OTUs (#8, #13, #18, and #37) which, based on their 16S, are related to *Micrococcus letues*. To save the compound, in the next steps, we only examined activity against OTU #13, which showed the greatest inhibition in the disk diffusion assay. The crude (DCM and MeOH) extract of bacterium cells had the strongest inhibition, followed by the MeOH extract of the agar and finally, the agar DCM extract.

**Figure 5:**
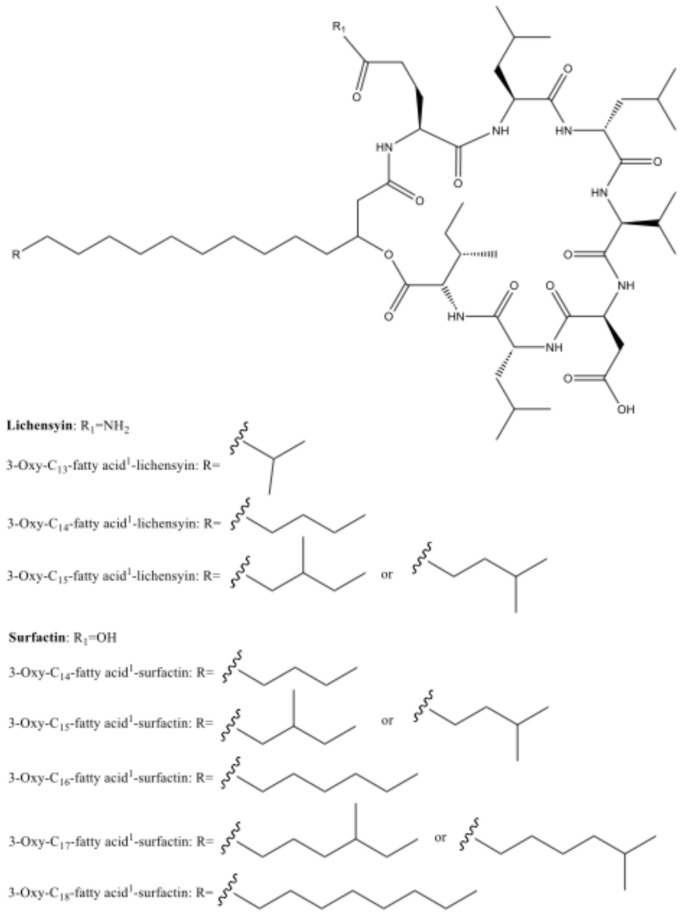
Lichenysins and surfactins from *Paenisporosarcina indica* associated with the marine sponge *Calyx nicaeensis*.

The crude extracts were separated via a size exclusion column. Fractions were re-combined based on the TLC pattern. Fractions 1, 2, 3, and 5 were active and merged based on composition similarities (by TLC and NMR). For further purification, a reversed-phase chromatograph was used. One of the eluted fractions from the cell mass and one from the agar extracts were found active (B-6, M-12). These active fractions were eluted from the column with high percentages of MeOH, indicating a compound(s) with hydrophobic characteristics. The active fractions were merged based on ^1^H NMR similarities. Fraction M-11 exhibited no activity; however, it displayed significant similarities to the active fractions in the ^1^H NMR and thus was further analyzed. Active fractions were purified and separated, resulting in three active fractions (5–7), along with two additional fractions (8–9) that appeared to contain similar compounds (but with partial activity). The separation of all these fractions was guided by antibacterial assays and monitoring of their ^1^H NMR spectra. Fraction 9 was found to be a pure compound and thus was spectroscopically analyzed. The other fractions were not pure but rather mixtures of related compounds and in minute quantities, preventing further separation. Fraction 9 constituted 0.092% of the crude extract. The crude extract of the bacteria cell mass was more active than that of the growth medium, suggesting that the active compounds are likely stored within the cells and are less expected to be secreted under these growth conditions. The fractions were active against the Gram-positive OTU #13, which is closely related to *Micrococcus luteus*, a potential pathogen. A summary of separations and obtained fractions is given in Figure S2, which presents the weight of active fractions following HPLC separation. The ^1^H NMR spectra of fractions 5–10 from HPLC separation revealed a group of compounds similar to known biosurfactants [54]. The various fractions presented molecular ions ([M-H]^−^) at *m*/*z* between 1000 and 1070 (Table 1). The compounds contain a fatty acid chain that contributes to their hydrophobic characteristics (Figure 5). The structure of the major component in major fraction 9 (24.4 mg) was elucidated through the analysis of the 1D (^1^H, ^13^C) and 2D (HSQC, HMBC, COSY, TOCSY, ROESY) NMR spectra (in Pyridine-*d*_5_) and HRMS (MW 1034 mu) and was found to be 3-oxy-*anteiso*-C_15_-fatty acid-lichenysin (Figures S3–S17). The length of the 3-oxy-fatty acid was deduced from the MS data, while the methyl branching point was deduced by interpretation of the NMR data.

## 3. Discussion

The main goal of this study was to investigate the bacterial community of the sponge *C. nicaeensis* as a potential source of natural products. Moreover, we specifically explored the sponge-associated bacteria interactions, aiming at identifying bacterial candidates that produce antibacterial compounds. To achieve this goal, we first cultured sponge-associated bacteria under various conditions. Sponge homogenization to cultivate and isolate bacteria may result in less bacterial diversity than expected. The observed relatively low diversity is due to dominant bacterial species prevailing in the culture conditions, outcompeting and suppressing the growth of less competitive ones. Greater diversity in the media and culture conditions typically results in a higher diversity of cultured bacteria, as previously demonstrated for sponge-associated bacterial communities [19,25].

In this study, four different media types with diverse carbon and nitrogen sources were used. In addition, a nalidixic acid antibiotic was applied, resulting in five different growth conditions overall. A total of 319 isolates from *C. nicaeensis* were eventually clustered into 90 OTUs and classified to their closest phylogenetic neighbor species (and strain) levels. The *C. nicaeensis* cultured OTUs belong to three phyla (Proteobacteria, Firmicutes, and Actinobacteria), which are frequently cultured from sponges [8]. The antibiotic was employed during the cultivation process with the following dual purpose: to enhance diversity by promoting the growth of bacteria with slower division rates while simultaneously encouraging the growth of the Gram-positive Actinobacteria. This choice was initially made due to Actinobacteria’s known ability to produce secondary metabolites [55]. The number of OTUs that grew on plates containing nalidixic acid was 1.38-fold lower than those cultured without antibiotics. This result is similar to values given in a recent review [21] that reported a ~1.5-fold lower richness. However, it does not appear that antibiotics have promoted the growth of Gram-positive bacteria. Bacteria that grew with or without antibiotics may differ due to slow/rapid division. OTUs cultivated solely on plates with antibiotics (n = 29) enhanced the overall diversity of obtained strains. Most OTUs grew on a single medium, which reflects their specific nutrition requirements. It is important to remember that culture efforts of most sponge-associated bacteria are unsuccessful [23,24,25].

The current study is novel in several aspects, as follows: 1. The choice of the sponge *C. nicaeensis* was critical because the sponge microbiome tends to be species-specific [8,11,56]. 2. The sampled sponge specimens were collected from the mesophotic region, which is an underexplored habitat, unlike shallow water (or terrestrial) sources, from which numerous antibacterial compounds have been discovered [57]. 3. It examined the identity of *C. nicaeensis*-associated microbiota. 4. The study tested interspecific interactions among the bacteria cultivated from the *C. nicaeensis*-associated bacteria. 6. Using ecologically relevant bacteria might uncover bioactive compounds that are missed by using standard laboratory strains [58]. 7. The research identified *Paenisporosarcina indica* as a producer of the biosurfactants lichenysins and surfactins. This is the first instance of a non-*Bacillus* bacterium being found to biosynthesize these compounds. 8. It is the first time that *P. indica* has been found in a marine environment and outside a cold ecosystem.

Moreover, the approach expressed in this study mitigates supply issues that may arise in the later stages of establishing a pharmacological production pipeline [34]. The approach lays the foundation for the biotechnological exploitation of the relevant OTUs, including possibilities for optimizing growth conditions and the genetic manipulation of bacterial producers.

Sponge-associated Gram-positive bacteria were chosen to be screened for antibiotic activity since Actinobacteria are known as proliferous secondary metabolite producers [59,60]. The chosen competition assay is a simple, fast, and low-cost assay that enabled the screening of 22 OTUs. This test showed the bacteria interspecific interactions under specific lab conditions. Active bacteria need to secrete substances from the cell in ample quantity and strength that will still be effective after the concentration decreases with distance from the source bacteria. Bacteria deemed non-active may secrete substances under different conditions or in a low concentration. The selected competition assay has a few disadvantages in choosing a bacterium for antibacterial compound extraction. It is not possible to know whether the inhibition is caused by a secreted active compound or because the test bacterium changes the conditions of the substrate (for example, altering pH, salt concentrations, etc.) or better utilizes nutrients from the medium. Another disadvantage is that different compounds have diverse diffusion rates, and the inhibition strength does not necessarily reflect the “real” strength of the active compound. Furthermore, since the tested bacterium does not come in contact with the competing bacteria, bacteria that anchor the antibacterial compounds in the cell wall without releasing them, may be overlooked by such an assay. The screening process was designed to detect negative (inhibition) and positive (growth promotion) interspecific interactions between Gram-positive bacteria against all OTUs grown on ISP2. ISP2 medium was selected because it supported the growth of the highest bacterial diversity. Gram-positive bacteria experienced greater growth inhibition than Gram-negative bacteria, whereas Gram-negative bacteria exhibited a higher degree of growth promotion than Gram-positive bacteria. The bacterium that inhibited the highest percentage of OTUs was OTU #72, inhibiting 22 out of 40 OTUs (Figure 3). The bacterium closely related molecularly to OTU #72 is *Paenisporosarcina indica*, with 99.14% similarity and 78.8% completeness. *Paenisporosarcina indica* was identified as a new species in 2013 when it was isolated from a glacier in the Himalayas but was never cultured from the marine environment [61]. The first report of *P. indica* as having a biotechnological potential for different traits was in 2020 [62]. However, antibacterial potential was not among the reported properties of this species. OTU #72 showed a strong inhibition >1 cm against OTUs #18, #37, and #22 (closest related bacteria: *Micrococcus luteus* (#18 and 37) and *Brevibacterium frigoritolerans* (#22)). Interestingly, OTU #72 inhibited OTU #69, which is closely related to *Paracoccus yeei*, a known opportunistic pathogen in humans [63]. The bacterium that showed inhibition against the second-highest number of OTUs (16) was OTU #10 (Figure 3). It is closely related to *Bacillus altitudinis*. OTU #10 also inhibited OTUs whose closest related bacteria are considered pathogens, such as OTU #69 (mentioned above), OTU #3—*Vibrio campbelli*—a marine aquaculture pathogen [64], and OTU #45—*Bacillus cereus*—a pathogen associated with food poisoning and severe eye infections [65]. Third in the hierarchy, with 14 inhibited OTUs, was OTU #43 (Figure 3). Its closest related bacterium, *Bacillus paralicheniformis*, is a known producer of an antibacterial compound against Gram-positive pathogenic bacteria such as *Staphylococcus aureus* [66]. OTU #43 also inhibited OTUs whose closest related bacteria are pathogens (e.g., OTU #69 and #45). The study revealed that a significant number of bacteria exhibited the ability to both inhibit OTUs and promote the growth of different coinciding bacteria. Notably, the capacity of some bacteria to inhibit strains closely related to pathogenic bacteria could offer promising opportunities for advancements in biotechnology and pharmaceuticals.

Inhibitory activity, as shown here, gives the host control over its residing microbiome; it may play a role in host protection from marine pathogens, as well as metabolically or chemically enhancing the competitive abilities of the hosting sponge. Inter-microbial antagonism, as seen in this research, has been described in previous studies that examined sponge-associated bacteria, e.g., [67,68]. We therefore anticipated and searched for inhibition interactions. Inhibitory effects in this study ranged from 0 to 55%. This is comparable to 0–70% found in a study of nine sponges [69]. The present study focused on a group of Gram-positive-associated bacteria cultivated on an ISP2 medium. Follow-up studies could consider that the number of antibacterial active sponge isolates can vary over time [70], suggesting a possible seasonal effect. Other factors influencing the proportion of antibacterial active bacteria obtained include the 1. Origin and Gram-type of test bacteria: the source of the test bacteria and their Gram staining characteristics can significantly impact antibacterial activity; Number of sub-cultivations: the number of times the bacteria are sub-cultivated before testing can affect their behavior and the outcomes of antibacterial assays; 3. Isolate diversity: the genetic and phenotypic diversity of bacterial isolates used in testing might influence the observed antibacterial activity; 4. Test method and conditions: variations in the methodology and conditions of the antibacterial assays (e.g., the medium, temperature, incubation time) can affect the results; 5. Competition assays: performing additional different competition assays against various pathogenic strains and OTUs may provide further insights into antibacterial activity; and 6. Inhibition patterns: generally, weak inhibition is more common, with strong, one-directional inhibition being rare. This latter observation coincides with a mathematical model predicting that weak and uncooperative inhibitions help stabilize diverse microbial communities [11]. Based on the results, a highly ranked candidate, OTU #72, was chosen for chemical extraction in order to check if it contains a potent antibacterial compound. OTU #72 cell mass and the culture medium (agar) were separately extracted (1:1 mixture of MeOH/DCM) and evaporated to crude extracts. The crude extracts were separated on a (Sephadex LH-20 column), followed by a TLC pattern to recombine similar fractions. The first fraction was active, indicating this is a relatively large compound. The MeOH and DCM agar fractions were merged due to similarities in their ^1^H NMR spectra. Further purification (EZPrep, reversed-phase C-18 column) indicated that one of the eluted fractions from the cell mass and one from the combined extractions were active (B-6 and M-12, respectively). The active fractions were eluted in high MeOH percentages, indicating a hydrophobic compound. The active fractions were merged based on ^1^H NMR similarities. Fraction M-11 exhibited no antibacterial activity; however, it displayed significant similarity to the active fractions in ^1^H NMR, thus further separated. The additional purification of M-12, B6, and M-11 (reversed-phase HPLC on a preparative C-18 column) resulted in three active fractions (5–7), along with two additional fractions (8–9) that appeared to belong to the same family (partially active). Fraction M-11 was similarly subjected to HPLC separation, yielding an additional fraction (8) belonging to the same family. However, it was not tested for activity due to limited quantity. All chromatographic separations were guided by antibacterial assays and ^1^H NMR spectra. Fraction 9 seemed to be a pure compound that was suitable for spectral analysis. The other fractions were not pure but rather mixtures of related compounds and in minute quantities preventing further separation. Fraction 9 constituted 0.092% of the crude extract. The crude extract of the bacteria cell mass was more active than the growth medium, suggesting that the active compounds are likely stored within the cells and are less expected to be secreted under these particular growth conditions. 

The fractions were active against the Gram-positive OTU #13, which is closely related to *Micrococcus luteus*—a potential pathogen. Most known antibiotics are active against Gram-positive bacteria, and one of the mechanisms of antibiotics is damaging the cell walls of Gram-positive bacteria. The latter have a thick peptidoglycan layer in their cell walls, which provides structural support, while Gram-negative bacteria have a thinner peptidoglycan layer surrounded by an outer membrane. This outer membrane acts as a barrier, making it more challenging for antibiotics to access the cell wall [71]. Gram-negative bacteria have multiple-drug efflux pumps that remove many antibiotics from their cells [72]. Another contributing factor to the higher susceptibility of Gram-positive bacteria is their slower growth rate. Bacteria are most vulnerable to antibiotics when they are actively growing and dividing. Slower-growing bacteria spend more time in this susceptible state, which can make them more responsive to antibiotic treatment [73].

Gram-positive bacteria can play a significant role in biofilm formation. Biofilms are complex communities of microorganisms that adhere to surfaces and are encased in a matrix of extracellular polymeric substances (EPS). Antibacterial activity against them may contribute to preventing the establishment of such biofilms, which have various applications, including inhibiting biofouling on ships and underwater structures [74]. Slow-diffusing compounds, such as those found in the current research, would be more favorable, as they would remain on the substrate for a longer duration and have less tendency to diffuse into the surrounding seawater. The initial spectroscopic analysis of fraction 9 indicated that the compound is a cyclic lipopeptolide containing a cyclic peptide/peptolide with a fatty acid sidechain. Many lipopeptides are both biosurfactants and antibiotics [75,76]. From HPLC B6 and M12, fractions 5, 6, 7, and 8 resemble isomers of surfactin, while fraction 8 from HPLC M11 looked like isomers of lichenysin (Table 1). The fractions may differ from each other by variations in their amino acid contents. The largest fraction 9 was found to contain one major isomer of lichenysin, 3-oxy-*anteiso*-C15-fatty acid-lichenysin (Table S1), which is a compound discovered by Li et al. (2010) [54]. The structures of the rest of the fractions were established based on their negative-HRESIMS and ^1^H NMR data, as follows: fraction 5—a mixture of surfactin/lichenysin with Glu^2^ and 3-oxy-C_14_-fatty acid^1^ (10%), 3-oxy-C_15_-fatty acid^1^ (66%), and 3-oxy-C_16_-fatty acid^1^ (24%); fraction 6—a mixture of surfactin/lichenysin with Glu^2^ and 3-oxy-C_13_-fatty acid^1^ (14%), 3-oxy-C_15_-fatty acid^1^ (21%), and 3-oxy-C_16_-fatty acid^1^ (65%); fraction 7—a mixture of surfactin/lichenysin with Glu^2^ and 3-oxy-C_13_-fatty acid^1^ (15%), 3-oxy-C_14_-fatty acid^1^ (12%), 3-oxy-C_15_-fatty acid^1^ (15%), 3-oxy-C_16_-fatty acid^1^ (24%), and 3-oxy-C_17_-fatty acid^1^ (34%); fraction 8—a mixture of lichenysins with 3-oxy-C_13_-fatty acid^1^ (5%), 3-oxy-C_14_-fatty acid^1^ (34%), and 3-oxy-C_15_-fatty acid^1^ (12%) and surfactin/lichenysin with Glu^2^ and 3-oxy-C_16_-fatty acid^1^ (38%) and 3-oxy-C_17_-fatty acid^1^ (10%); and fraction 10—3-oxy-*iso-*C_15_-fatty acid-lichenysin (86%) and surfactin/lichenysin with Glu^2^ and 3-oxy-C_16_-fatty acid^1^ (14%).

Lichenysin and surfactin are bioactive cyclic lipopeptide secondary metabolites and, so far, are known to be produced solely by bacteria belonging to the genus *Bacillus*. In this study, we demonstrated the production of lichenysin, surfactin, and related compounds by a bacterium that belongs to another genus, *Paenisporosarcina*, from the same order as *Bacillus*.

Surfactins and lichenysins constitute a group of very effective biosurfactants with many known pharmacological effects, such as antibacterial, antifungal, antiviral, and other activities [77,78,79,80]. Since biosurfactants are effective in reducing surface tension, they are key targets for research focusing on developing novel antimicrobial agents due to their biological activities [81]. Both biosurfactants share a common structure comprising a hydrophilic peptide ring with seven amino acids. This ring is linked to a hydrophobic β-hydroxy fatty acid chain, with a length that ranges from 12 to 17 carbons [82,83]. Lichenysin differs from surfactin in the first amino acid, which is glutamine instead of glutamic acid [76]. Additionally, various isomers of both compounds often coexist, resulting from a combination of different peptide variations and various aliphatic chain lengths [84,85]. The various isomers share similar features but can differ in physicochemical and bioactive properties [86,87,88]. While separating the compounds using reversed-phase HPLC, we noticed that the compounds strongly bind to the C-18 column, which resembles certain membranes in their hydrophobic nature. We assume the compounds’ fatty acid chain has a strong interaction with the C-18 column, and therefore, its elution is delayed. Both lichenysin and surfactin are known to destabilize and disrupt membranes using their amphiphilic characteristics [80]. The hydrophobic fatty acid chain penetrates the membrane, followed by structural changes, which contribute to the interaction process [89]. This binding has been demonstrated in vitro to induce the dehydration of the phospholipid polar head group. It therefore severely affected the bilayer’s stability, leading to disruptions in the membrane barrier properties. Such a mechanism may explain the antibacterial effects [90]. 

This study demonstrated low production values of the compounds analyzed, which resemble the challenges in the industrial production of such biosurfactants. The industrial synthesis of these compounds often suffers from high costs and low yields [91]. In this research, two types of powerful lipopeptides—two lichenysins and four surfactins—were successfully co-produced from *P. indica* cultivated on the ISP2 medium. This dual production, which had been previously unreported for *P. indica*, opens new possibilities for biosurfactant production. Future investigations could explore optimizing growth conditions for *P. indica* to enhance the yield of these valuable compounds.

It was thus shown that culturing sponge-associated microorganisms under various conditions increases the likelihood of detecting new producers and novel compounds with bioactive properties. In this study, the activity of compounds evolved through natural interspecific interactions among associated microorganisms. The results illuminate the potential for harnessing these natural interactions in the development of new pharmaceuticals.

## 4. Materials and Methods

### 4.1. General Experimental Procedure

Optical rotation values were obtained on a Jasco P-1010 polarimeter at the sodium D line (589 nm). UV spectra were recorded on an Agilent 8453 spectrophotometer. IR spectra were recorded on a Bruker Tensor 27 FT-IR instrument. NMR spectra were recorded on Bruker Avance III spectrometers at 500.13 MHz for ^1^H and 125.76 MHz for ^13^C, and chemical shifts were referenced to TMS δ_H_ and δ_C_ = 0 ppm. DEPT, COSY-45, gTOCSY, gROESY, gHSQC, and gHMBC, spectra were recorded using standard Bruker pulse sequences. ESI low- and high-resolution mass spectra and MS/MS spectra were recorded on a Waters (USA) Xevo G2-XS QTOP instrument equipped with Acquity Hi class UPLC (binary solvent manager) with an FTN sample manager, column manager, and PDA detector using a 2.1 × 50 mm BEH C18 (1.7 μm) column and a flow of 0.1–0.3 mL/min. HPLC separations were performed on an Agilent 1100 Series HPLC system. HPLC separations were performed on a Merck Hitachi HPLC system (L-6200 Intelligent pump and L-4200 UV-VIS detector), a JASCO P4-2080 plus HPLC system with a multiwavelength detector, and an Agilent 1100 Series HPLC system.

### 4.2. Sample Collection

Two specimens of the sponge *C. nicaeensis* were collected from Yam-Poleg, Israel (32°10.41 N, 34°37.84 E) at a ~100 m depth (with permit 2021/42929 from Israel Nature and National Parks Protection Authority). One specimen was collected on 24 November 2021, and another specimen was collected on 27 February 2022. The specimens were collected using a remotely operated vehicle (ROV; ECA-Robotics H800) equipped with a five-function manipulator, a high-definition camera, and two parallel laser beams for scale reference. To safely retrieve the selected sponge, it was placed in a basket and brought to the research vessel “Mediterranean Explorer”, operated by EcoOcean. The collected specimens were photographed, divided, and processed for different downstream analyses. One subsample was preserved in 85% ethanol and deposited as a voucher in the Steinhardt Museum of Natural History, Tel Aviv University. A second subsample was immersed in cold seawater to maintain stable pH and salinity, thereby preserving the sponge and its associated microbiome for microbial cultivation. The third subsample was frozen at –20 °C onboard and later transferred to –80 °C in the laboratory for subsequent cultivation from frozen samples.

### 4.3. Cultivation of C. nicaeensis—Associated Bacteria

The two hosting sponges were transported to the laboratory and processed on the collection day to maintain sponge and microbiome viability. A small piece (~7 cm^3^) containing the ectoderm and endoderm of each specimen underwent the following procedure: To eliminate epibiont and transient bacteria, sterile calcium–magnesium-free artificial seawater (CMF) solution was used for rinsing the specimens (containing NaCl 26.24 g/L, Na_2_SO_4_ 4.69 g/L, KCl 0.67 g/L, NaHCO_3_ g/L, and Na EDTA 0.37 g/L, pH 8.2). Subsequently, the samples were homogenized with 44 mL of CMF in a blender and transferred to 1.5 mL test tubes after filtration through a 50 µm mesh. The procedure was performed under sterile conditions within a laminar flow hood. Three dilution factors (100, 10^−1^, 10^−2^, and 10^−3^) were prepared with CMF, and 100 µL of each dilution was inoculated onto the following four different types of media in duplicates: SCA, MA, ISP2, and LB (details in Table S2). The use of various media containing various nutrients was aimed at increasing the diversity of isolated bacteria [92]. The LB medium contained antibiotics, while ISP2 was prepared with and without antibiotics (10 µg mL^−1^ nalidixic acid, targeting Gram-negative bacteria). The antibiotic was used to selectively cultivate Gram-positive bacteria, such as Actinobacteria [54]. All media were supplemented with 100 µg mL^−1^ of cycloheximide to prevent fungal growth and adjusted to a pH of 8.2 and salinity of 39 ppt, resembling *C. nicaeensis* natural seawater conditions. Antifungal agents and antibiotic solutions were sterilized using a 0.22 µm filter. Following inoculation, the plates were incubated at 25 °C in the dark to avoid the degradation of the antibiotics.

### 4.4. Morphological Characterization of Bacterial Isolates

The incubation of cultivated plates spanned four weeks, during which regular inspections were conducted every few days. Colonies displaying distinct morphological characteristics across all media and duplicates, such as color, shape, texture, diameter, and surface area, were carefully noted, marked, and transferred to fresh plates using an isolation streak to obtain pure colonies. A second isolation streak was performed four days later to ensure the isolate’s purity. The resulting pure isolates were preserved in glycerol stock (25%) and 100% ethanol for future analyses.

### 4.5. Molecular Identification Using rDNA 16S Phylogenetic Analysis

Each isolate underwent DNA extraction. The isolates were suspended in 20 µL of ultra-pure water (UPW), heated at 90 °C for one minute, and then frozen at −20 °C. The application of thermal shock resulted in the disruption of the cell wall and membrane, facilitating the DNA extraction. The extracted DNA was then used to amplify approximately 1300 bp of the 16S rDNA region using universal primers 63F and 1387r [93]. The PCR reaction mixture and cycling parameters are provided in Table S3. The PCR products were separated using electrophoresis on a 1% agarose gel using 80V for 110 min and viewed under ultraviolet light. Amplified samples were sequenced with the forward primer 63f (Microsynth Seqlab, Göttingen, Germany). The raw sequence data were manually analyzed to ensure quality control and validation of automatic results using Geneious software (Geneious Prime 2021.0.1). The sequences were then clustered into (OTUs) using specific parameters, including a maximum of 3% gaps per read, a maximum gap size of 5, a minimum overlap identity of 97%, a word length of 10, an index word length of 10, a maximum of 1% mismatches per read, and a maximum ambiguity of 16. The consensus sequences of each OTU were compared with the EZtaxon database [94] to identify the closest related known bacterium. The consensus sequences, along with their closest relatives identified through EZtaxon and an outgroup 16S sequence of *Flavobacterium bizetiae* from the Bacteroidetes phylum, were aligned and trimmed. Maximum likelihood phylogenetic trees were constructed to analyze the phylogenetic relationships using MEGA software version 11.0.13 [95,96]. The program determined the best model for each phylum, considering a proportion of invariable sites (I) and a gamma distribution of site heterogeneity (G), with four gamma rates derived from the dataset. The phylogenetic analysis was supported by 500 bootstrap repetitions [97].

### 4.6. Competition Assay—Antibacterial Screening

**Figure 6:**
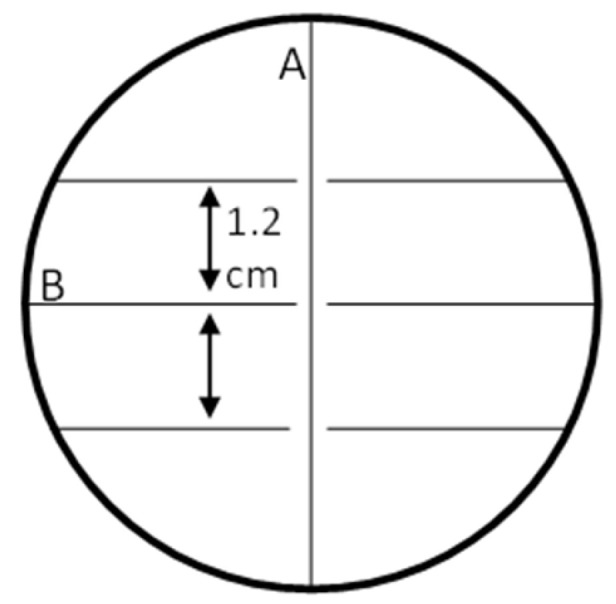
A schematic representation of a competition assay. A—The tested bacterium along the center line. B—Different OTUs smeared vertically with 1.2 cm between them, without touching the vertical line.

To test the bacterial interspecific interactions between the cultured symbionts and identify those exhibiting potential for producing antibacterial compounds, a competition assay was conducted. Because Actinobacteria are Gram-positive and known for their rich secondary metabolite arsenal, Gram-positive bacteria that grew on the ISP2 medium were preliminarily screened against all OTUs (both Gram-positive and Gram-negative) that grew on the same medium. This screening was performed without replicates to narrow down the initial laboratory work, a decision that was made due to the large number of tested OTUs. In the competition assay, the tested bacterium was inoculated along the center line of a Petri dish and incubated for four days. Then, cultured bacteria from each OTU were placed vertically near the middle line without physical contact with the tested bacterium, with 1.2 cm between them (Figure 6). All bacterial cultures were derived from 10 µL of liquid medium after being incubated overnight at 30 °C [37,98]. The test plates were examined after 24 and 48 h. A positive control group was incorporated as a reference for observing the typical growth of the tested bacterium [37]. A negative control was performed by inoculating a liquid medium devoid of any bacteria. Strains demonstrating the inhibition or stimulation of growth were subjected to experiments (in triplicates) to confirm their interspecific behavior. Strains exhibiting the strongest inhibition effect in the triplicate experiments against a broad range of bacteria were selected as candidates for compound extraction. Eigenvector centrality was calculated to help identify and rank the most significant bacteria within the inhibition network [99]. This measure is used in network analysis to determine the importance of nodes (in this case, bacterial species) based on both their direct connections and the importance of the nodes they are connected to. A high eigenvector score indicates strong connections to highly influential nodes. This ranking can highlight which bacterium has the most significant impact within the network, guiding the selection of which OTU is the “strongest” competitor to advance for chemical extraction and isolation of antibacterial compounds [99].

### 4.7. Antibacterial Guided Fractionation

After finding the “strongest” competitor, a mass culture of the bacterium and its culture medium were extracted in order to isolate the active component(s). The activity of the extracts was tested using disk diffusion assays [100]. The active fractions were separated using a Sephadex LH-20 Size Exclusion column, EZPrep reversed-phase MPLC column, and reversed-phase high-performance liquid chromatography (HPLC), to obtain a pure compound. After every separation step, a disk diffusion activity test was performed in order to validate that the activity was not lost and to continue with active fractions. Finally, the structure of the pure compound was determined using nuclear magnetic resonance (NMR) and mass spectrometry (MS).

### 4.8. OTU #72 Mass Production for Compound Extraction

Mass production of a specific bacterium is often necessary to obtain a sufficient quantity of the target compound for further analysis, research, and potential applications. OTU #72 was thawed from glycerol stock and inoculated onto ISP2 medium for a 72 h incubation. ISP2 agar medium was selected as the culture medium because the isolate originally grew best on this medium. For mass production, the bacteria were cultured using aluminum plates [101]. Initially, 60 plates were utilized, with each plate containing 230 mL of ISP2, resulting in 13.8 L of culture medium. Due to low quantities of the active compound, a second mass production was performed utilizing 80 plates, each containing 300 mL, resulting in 25.55 L. The bacteria were inoculated in streaks, with each streak containing 50 µL. The cultures were extracted by scraping the bacteria and cutting the medium into 2 cm^3^ pieces, followed by submerging them separately for 24 h in methanol/dichloromethane 1:1 (MeOH:DCM) under gentle shaking, followed by a second extraction with the same solvent mixture for 4 h. Both extracts were filtered (Whatman filter no.595 1/2) and evaporated using a rotavapor and gentle heating (<40 °C). In order to remove solid agar residues, the crude extract was dissolved again in MeOH:DCM and filtered. The extract was tested for antibacterial activity. The first culture yielded 11 g of crude extract, and the second, in which the MeOH and DCM fractions were separated, yielded 11.64 g and 3.86 g, respectively. Additionally, bacteria extracts were further processed in the second round with a MeOH:DCM ratio of 1:1, resulting in 3.42 g of extract. Extracts were weighed and stored at 4 °C until use in the assay.

### 4.9. Antibacterial Activity Disk Diffusion Assay from the Mass Production of OTU #72

**Figure 7:**
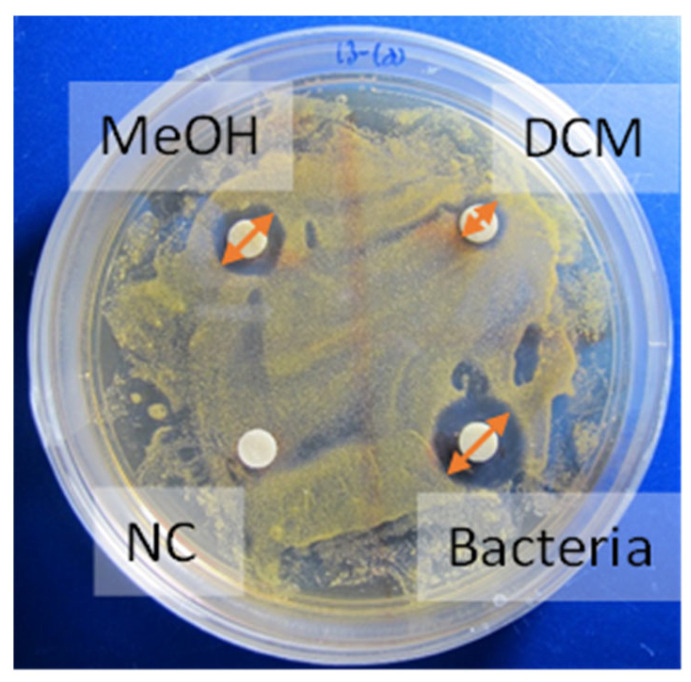
Disk diffusion assay. MeOH, DCM, and bacteria cell mass extracts on filters. NC—negative control. Extracts show antibacterial activity (halo), while the NC does not.

The crude extracts were dissolved in methanol/dichloromethane 1:1 to a final concentration of 100 and 50 mg/mL, and 15 µL were placed on sterile paper disks in triplicates and dried to minimize the potential effect of the solvent [100]. Liquid media (100 µL) of different bacteria (post-24 h incubation with 50 rpm) were inoculated on an ISP2 agar plate. The bacteria were spread on the agar with T-shaped spreaders and allowed to dry for one hour before the paper disks were placed (Figure 7). The plates were wrapped with parafilm and incubated at 30 °C. Inhibition zones were documented after 24 and 48 h and examined using ImageJ (version 1.53 packaged for macOS with Java). A negative control paper disk was placed, having only MeOH:DCM 1:1. Negative plates were performed to ensure bacteria growth.

### 4.10. Chromatographic Separation Methods

The crude extract was separated using four chromatographic steps in order to isolate the antibacterial active compound(s). Initially, a size exclusion separation was conducted using a Sephadex LH20 column with MeOH/CHCl_3_ (1:1). The fractions were evaporated and analyzed using thin-layer chromatography (TLC). Based on the results, fractions containing a similar mixture of compounds were combined, and their ^1^H NMR was measured. The active fractions were then dissolved in methanol and separated based on polarities (EZPrep, RediSep^®^ reverse phase C18 column). The mobile phase was a gradient of solvents starting with 100% H_2_O and increasing amounts of MeOH, and then 100% ethyl acetate at a flow rate of 20 mL/min and monitored using UV at 214 nm and 254 nm. Finally, the active fractions B-6 and M-12 were merged based on ^1^H NMR similarity and separated on the preparative C-18 HPLC column. The sample was dissolved in MeOH at a concentration of 50 mg/mL and injected at a volume of 400 µL. The separation conditions were 90:10 ACN:H_2_O, with a flow rate of 5 mL/min, and monitored at a wavelength of 210 nm, resulting in nine fractions + waste. Based on the ^1^H NMR similarity of fraction M-11 to fractions B-6 and M-12, the fraction was further separated using the same column, concentration, flow rate, and wavelength. The fraction was injected at a volume of 200 µL. Separation conditions were 50:50 ACN:H_2_O. All fractions were tested for biological activity, as stated above.

### 4.11. Structure Elucidation of the Major Component of Fraction 9

In order to determine the chemical structure of the pure compound, several methods were combined. 1D ^1^H and ^13^C NMR spectra were measured in pyridine-*d*_5_, followed by 2D experiments COSY, TOCSY, ROESY, HMQC, and HMBC. In addition, HRESIMS and MS/MS were conducted.

## Figures and Tables

**Table 1:** MS of active fractions from the HPLC separation of extracts of OTU #72 bacteria cell mass.

HPLC of Fraction	Fraction	Weight (mg)	(-)HR ESI MS *m*/*z* (%)	Identity
M-12 and B-6	5	10.2	1020.6819 (8.3)	3-oxy-C14-fatty acid1-surfactin
			1034.6986 (57.8)	3-oxy-C15-fatty acid1-surfactin
			1048.7141 (21.5)	3-oxy-C16-fatty acid1-surfactin
			1051.7315 (12.3)	unknown
	6	12.8	1005.6753 (8.2)	3-oxy-C13-fatty acid1-lichensyin
			1034.6908 (20.4)	3-oxy-C15-fatty acid1-surfactin
			1048.7079 (60.8)	3-oxy-C16-fatty acid1-surfactin
			1065.7411 (10.3)	unknown
	7	7.5	1005.6635 (12.7)	3-oxy-C13-fatty acid1-lichensyin
			1019.6785 (10.0)	3-oxy-C14-fatty acid1-lichensyin
			1034.6807 (12.1)	3-oxy-C15-fatty acid1-surfactin
			1048.7025 (20.9)	3-oxy-C16-fatty acid1-surfactin
			1062.7135 (29.4)	3-oxy-C17-fatty acid1-surfactin
			1065.7251 (11.0)	unknown
			1076.7235 (3.0)	3-oxy-C18-fatty acid1-surfactin
	8	8.9	1005.6625 (5.8)	3-oxy-C13-fatty acid1-lichensyin
			1019.6788 (33.7)	3-oxy-C14-fatty acid1-lichensyin
			1034.6793 (12.0)	3-oxy-C15-fatty acid1-surfactin
			1048.6937 (38.3)	3-oxy-C16-fatty acid1-surfactin
			1062.6959 (11.0)	3-oxy-C17-fatty acid1-surfactin
	9	24.2	1005.6644 (2.4)	3-oxy-C13-fatty acid1-lichensyin
			1019.6813 (6.1)	3-oxy-C14-fatty acid1-lichensyin
			1033.6973 (71.5)	3-oxy-C15-fatty acid1-lichensyin
			1048.6980 (13.9)	3-oxy-C16-fatty acid1-surfactin
			1062.7126 (2.4)	3-oxy-C17-fatty acid1-surfactin
	10	1.0	1019.6771 (4.1)	3-oxy-C14-fatty acid1-lichensyin
			1033.6932 (80.2)	3-oxy-C15-fatty acid1-lichensyin
			1048.6935 (12.3)	3-oxy-C16-fatty acid1-surfactin
			1062.7090 (2.7)	3-oxy-C17-fatty acid1-surfactin
M-11	8 *	4.7	1019.6756 (86.9)	3-oxy-C14-fatty acid1-lichensyin
			1033.6915 (13.2)	3-oxy-C15-fatty acid1-lichensyin

* Was not active, however, based on ^1^H NMR its structure is similar to the other fractions family.

## Data Availability

The original contributions presented in this study are included in the article/Supplementary Material; further inquiries can be directed to the corresponding author/s.

## Supplementary Materials

The following supporting information can be downloaded at https://www.mdpi.com/article/10.3390/md22100440/s1: Figure S1. Phylogenetic tree A-D; Figure S2. Flow chart of extraction and chromatographic separations; Figures S3-S8 HRESIMS of fractions 5-10; Figure S9. HRESIMS of fraction M-11-8; Figure S10. ^1^H NMR (500 MHz) spectrum of fraction 9 in Pyr-*d*_5_; Figure S11. ^13^C NMR (125 MHz) spectrum; Figure S12. HSQC spectrum of fraction 9 in Pyr-*d*_5_; Figure S13. HMBC spectrum; Figure S14. COSY spectrum of fraction 9 in Pyr-*d*_5_; Figure S15. TOCSY spectrum of fraction 9 in Pyr-*d*_5_; Figure S16. ROESY spectrum of fraction 9 in Pyr-*d*_5_; Figure S17. Structure and key HMBC correlations of the major component of fraction 9; Table S1. NMR data of the major component of fraction B-6+M-12-HPLC-9 in Pyr-*d*_5;_ Table S2. Different medium compositions used to cultivate *C. nicaeensis-*associated bacteria; Table S3. PCR reaction mixture and cycling parameters of 16S amplification of *C. nicaeensis* associated bacteria.
