# Supplementary material for: Diversity and Activity of Bacteria Cultured from a Cup—The Sponge Calyx nicaeensis
Source: Mar Drugs. 2024 Sep 26;22(10):440. doi: 10.3390/md22100440 (PMC11509412; doi:10.3390/md22100440)
Supplement: Supplementary file 1 [file marinedrugs-22-00440-s001.zip › marinedrugs-3108231-supplementary.pdf]

Diversity and activity of bacteria cultured from a cup – the sponge *Calyx nicaeensis*

Lynne Itelson[1], Mayan Merav[2], Shai Haymi[1], Shmuel Carmeli[2], Micha Ilan[1]

[1] School of Zoology, Faculty of Life Science, Tel Aviv University, Tel Aviv 6997801, Israel; E-Mails: lynneitelson@hotmail.com (L.I.); milan@tauex.tau.ac.il (M.I.); shai12132@gmail.com (S.H.)

2 School of Chemistry, Raymond and Beverly Sackler Faculty of Exact Sciences, Tel Aviv University, Tel Aviv 6997801, Israel; E-Mails: mayanmv@gmail.com (M.M.); carmeli@tauex.tau.ac.il (S.C.)

* Correspondence: E-Mail: milan@tauex.tau.ac.il; lynneitelson@hotmail.com;

Supplementary Materials

Table of Content

Page	Title
2	Table of Content
3	Figure S1. Phylogenetic tree A
4	Figure S1. Phylogenetic tree B
5	Figure S1. Phylogenetic tree C
6	Figure S1. Phylogenetic tree D
7	Figure S2. Flow chart of extraction and chromatographic separations
8	Figures S3+S4 HRESIMS of fraction 5 & 6
9	Figures S5+S6 HRESIMS of fraction 7 & 8
10	Figures S7+S8 HRESIMS of fraction 9 & 10
11	Figure S9. HRESIMS of fraction M-11-8. Figure S10. ^{1}H NMR (500 MHz) spectrum of fraction 9 in Pyr-d_5.
12	Figure S11. ^{13}C NMR (125 MHz) spectrum & Figure S12. HSQC spectrum of fraction 9 in Pyr-d_5.
13	Figure S13. HMBC spectrum % Figure S14. COSY spectrum of fraction 9 in Pyr-d_5.
14	Figure S15. TOCSY spectrum & Figure S16. ROESY spectrum of fraction 9 in Pyr-d_5.
15	Figure S17. Structure and key HMBC correlations of the major component of fraction 9.
15-19	Table S1. NMR data of the major component of fraction B-6+M-12-HPLC-9 in Pyr-d_5
20	Table S2. Different medium compositions used to cultivate *C. nicaeensis*-associated bacteria.<br>Table S3. PCR reaction mixture and cycling parameters of 16S amplification of *C. nicaeensis* associated bacteria.

Figure S1. Phylogenetic trees from partial 16S rDNA sequences from bacteria isolated from *C. nicaeensis* and their closest related bacteria as inferred from EzTaxon database. Each tree represents a cultivated phylum – **A.** Actinobacteria **B.** Firmicutes **C.** Proteobacteria from the Alphaproteobacteria class **D.** Proteobacteria from the Gammaproteobacteria class. The evolutionary relations were inferred by using the Maximum likelihood method and a correct nucleotide substitution model was chosen for each tree. *Flavobacterium bizetiae* was used as an outgroup. Bootstrap values are based on 500 repetitions given at the nodes. The phylogenetic tree was constructed and visualized using MEGA (version 11.0.13).

A

Tamura-Nei model G+I

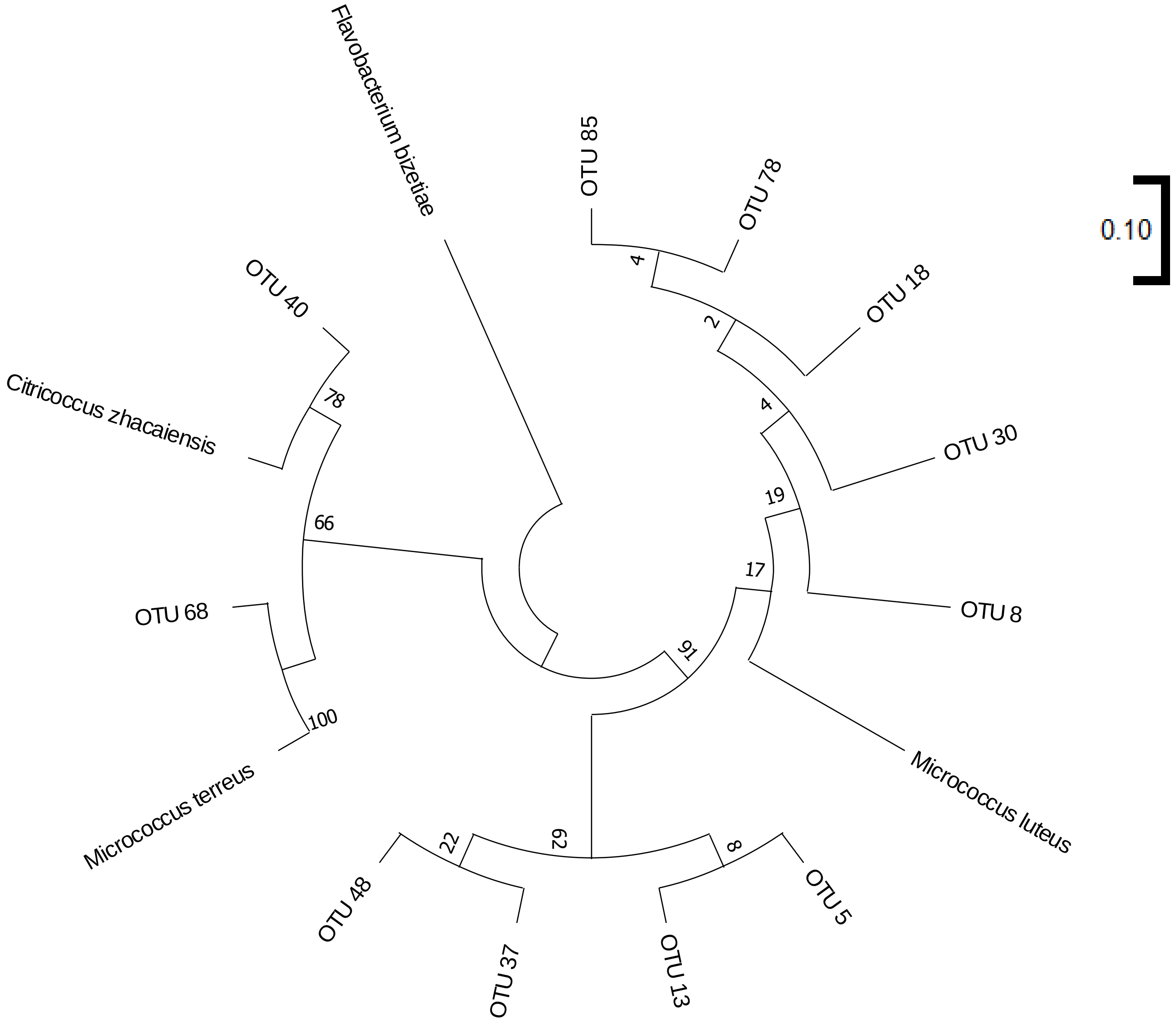

B

Kimura 2-parameter model G+I

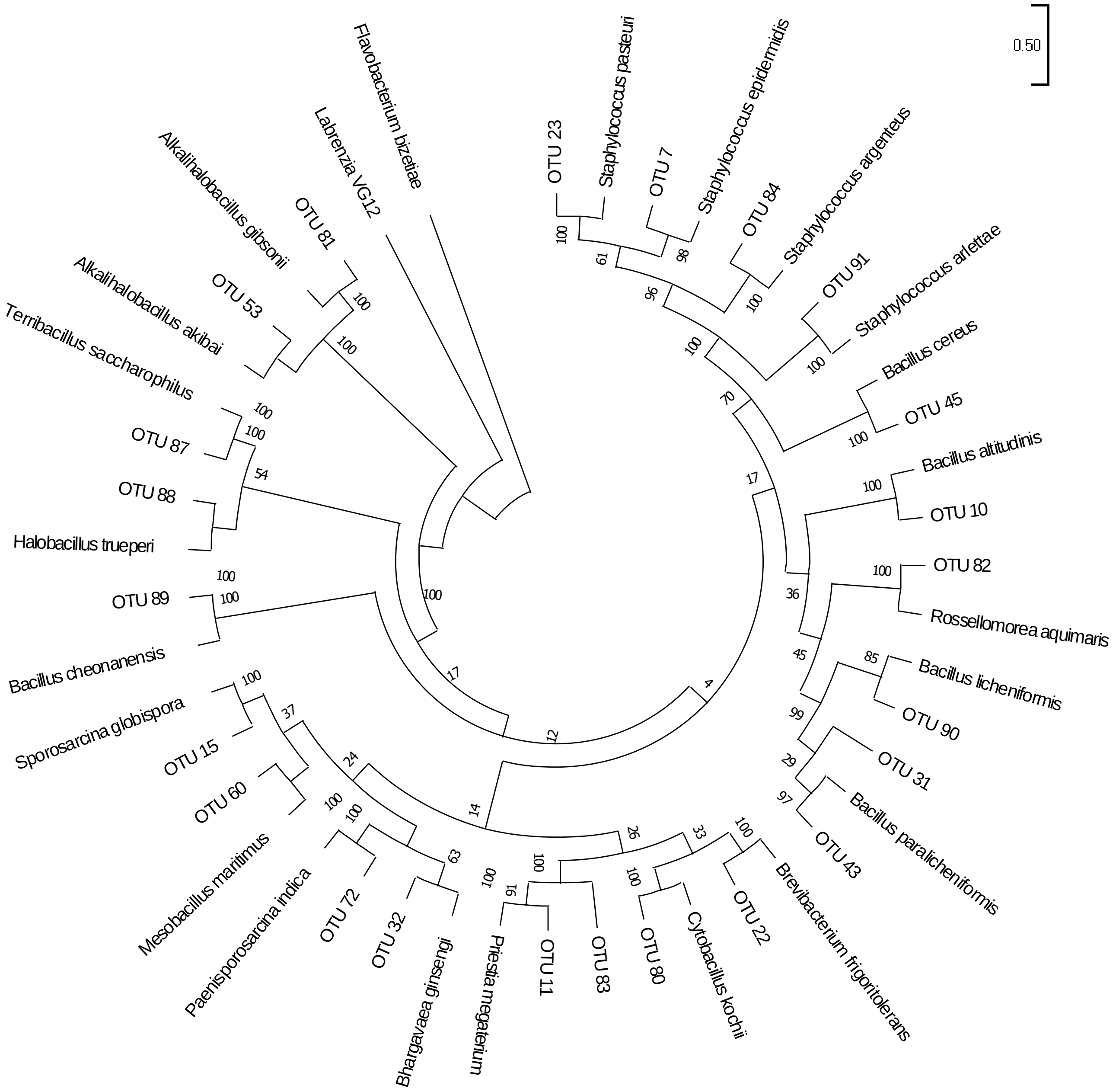

C

Kimura 2-parameter model G+I

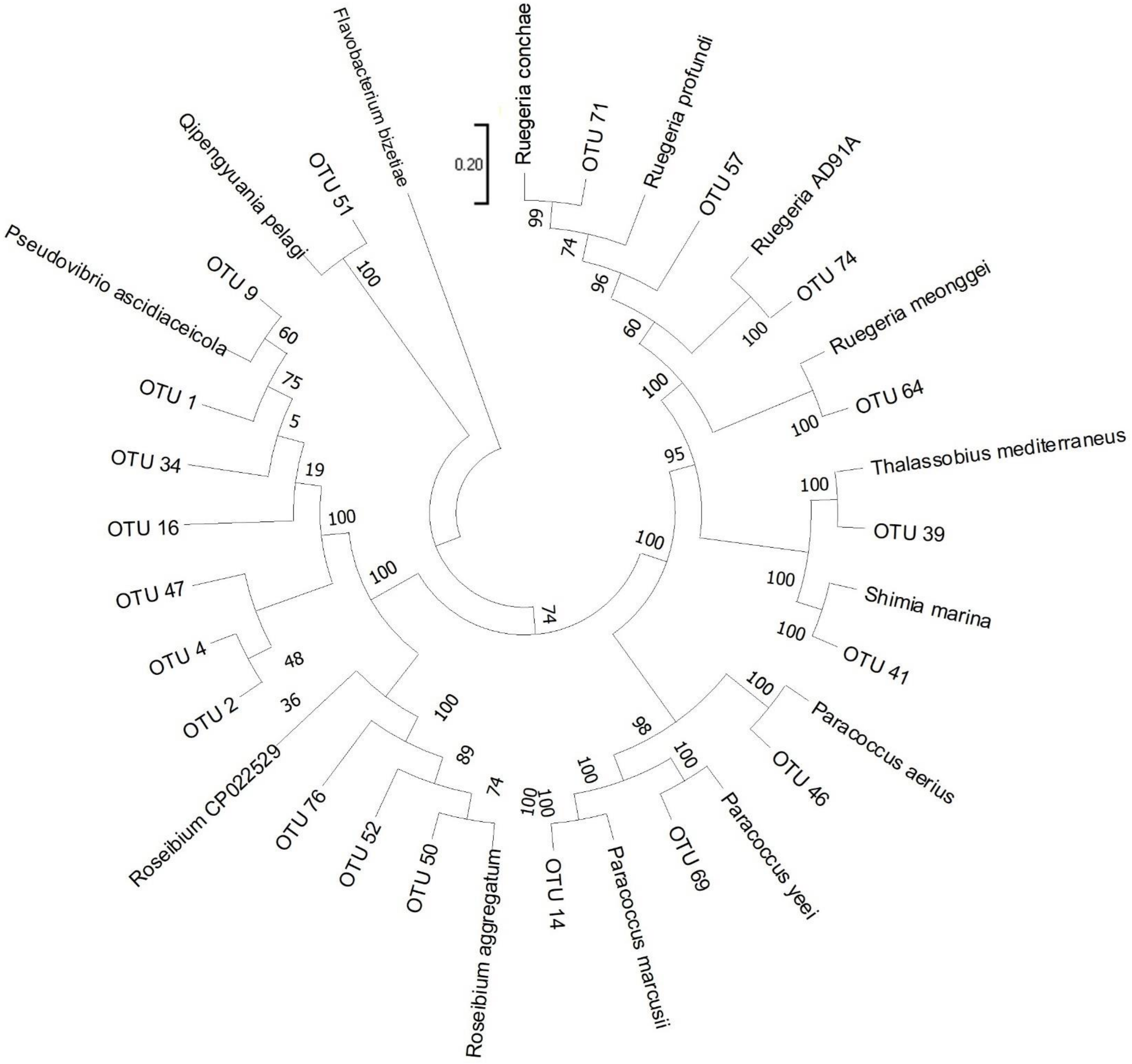

D

Kimura 2-parameter model G+I

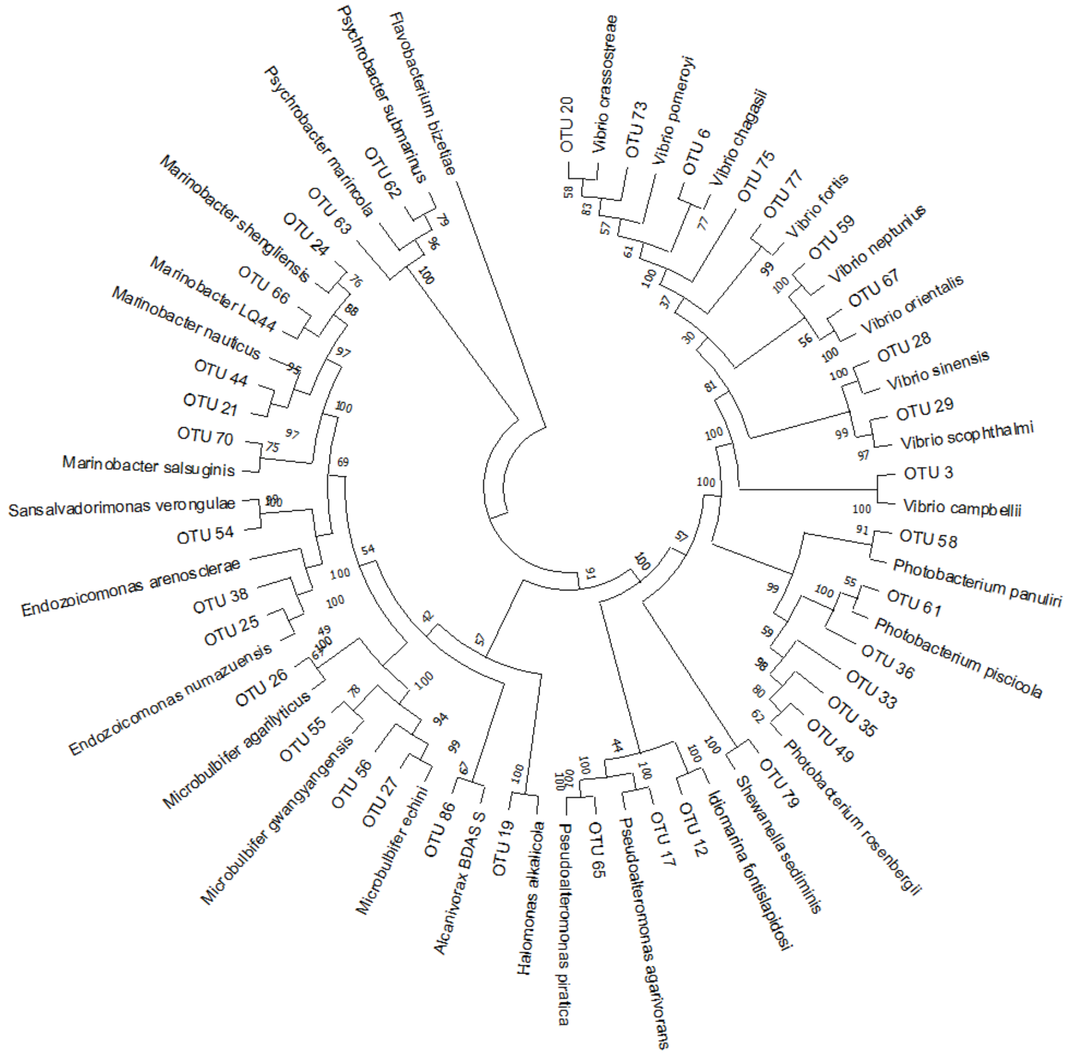

Figure S2. A flow chart describing the extraction and chromatographic separations on OTU #72 from the second extraction of 25.25 L. The weight of active fractions combined is given in every box. Weight of active fractions after HPLC is in Table 1.

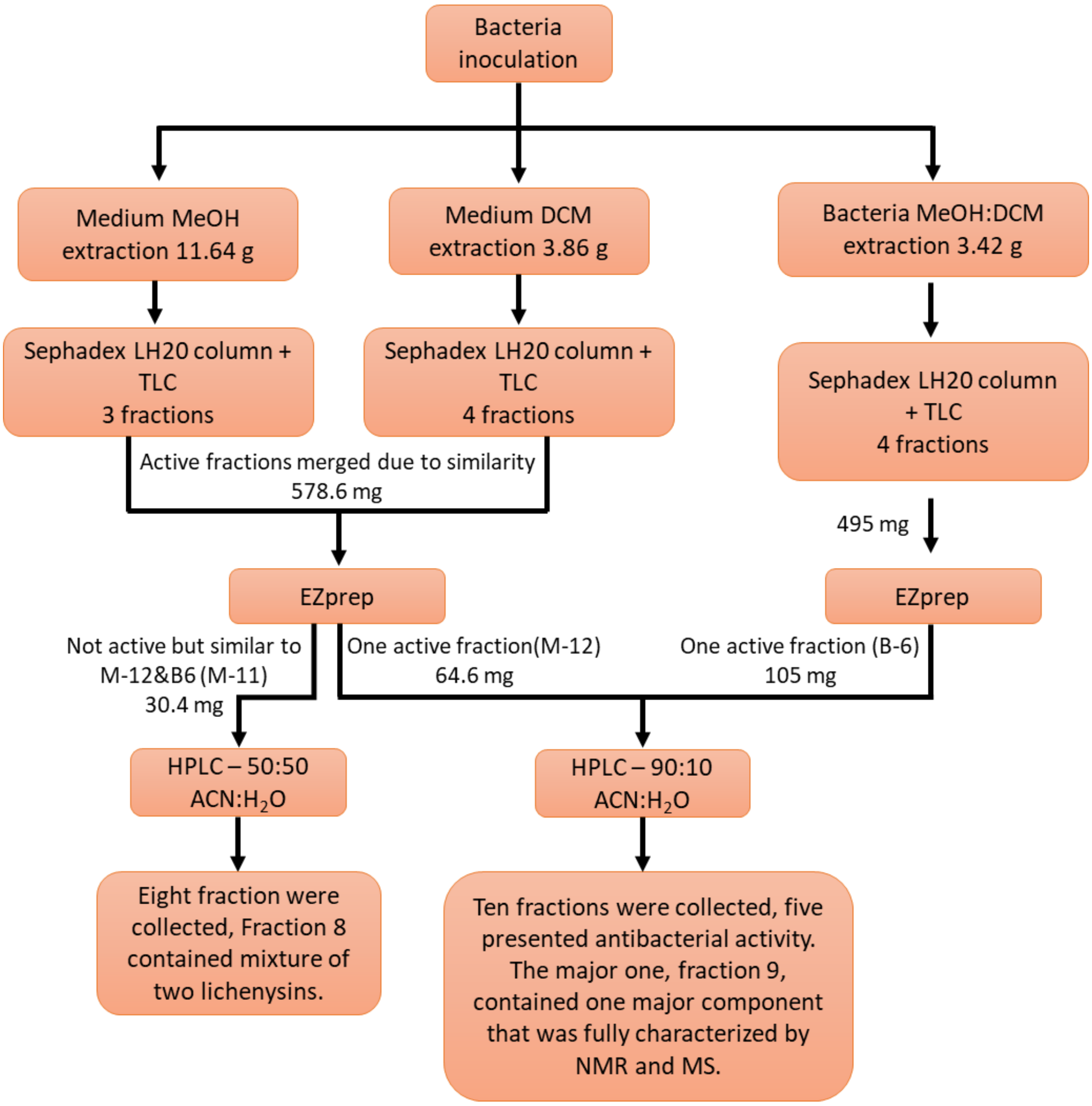

Figure S3. HRESIMS of fraction 5.

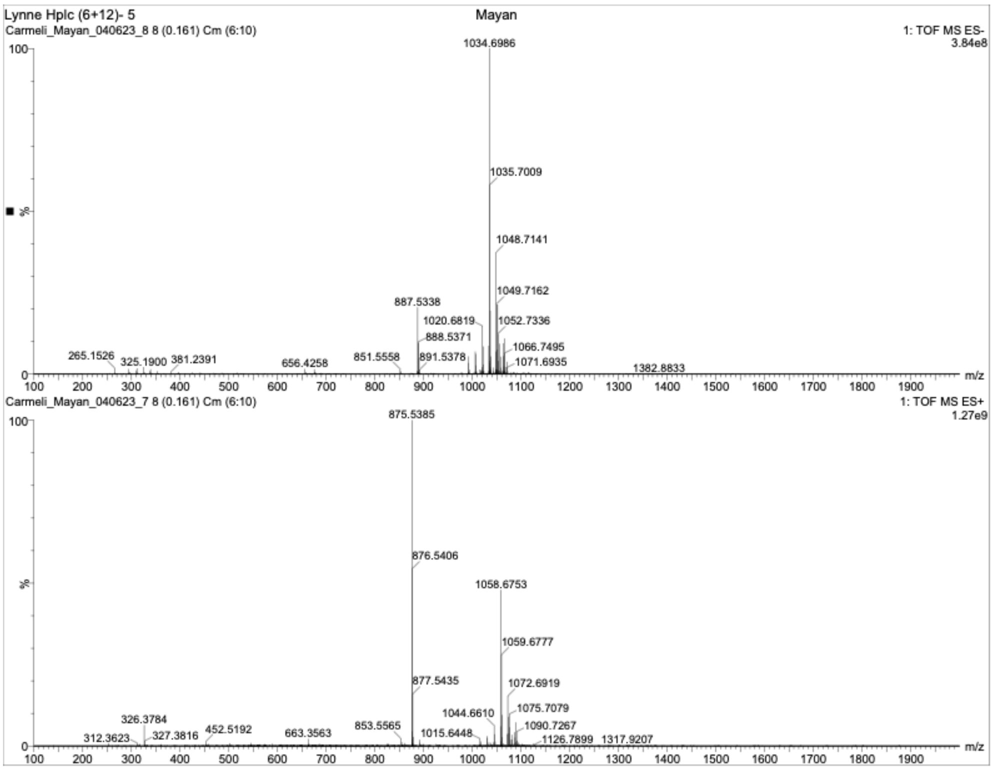

Figure S4. HRESIMS of fraction 6.

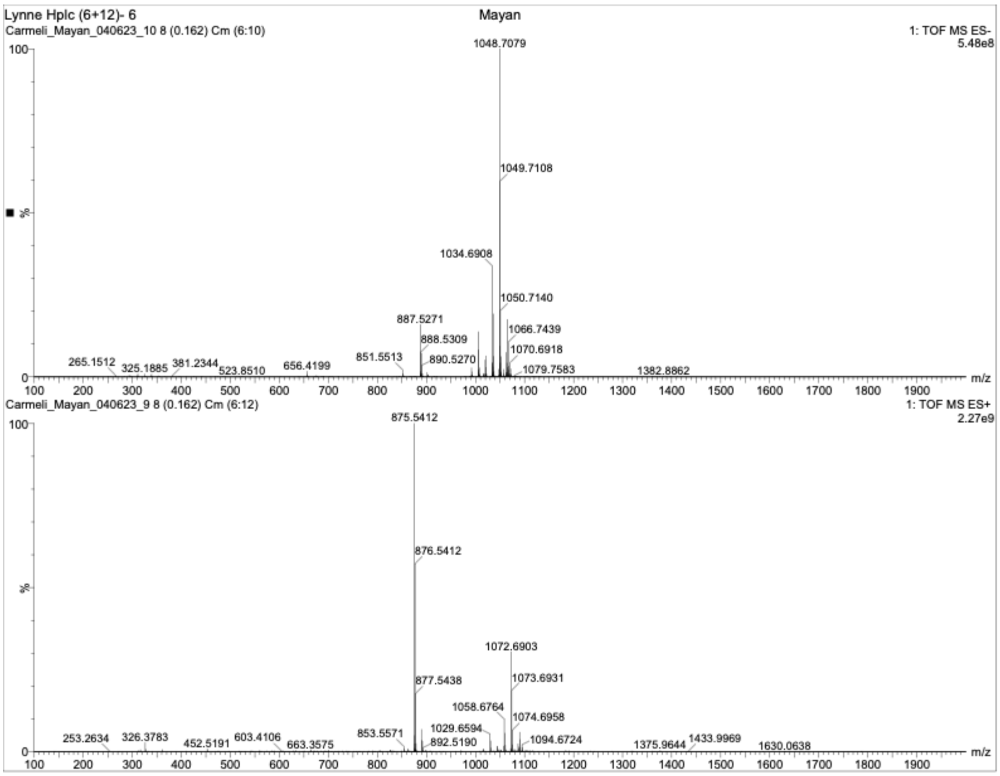

Figure S5. HRESIMS of fraction 7.

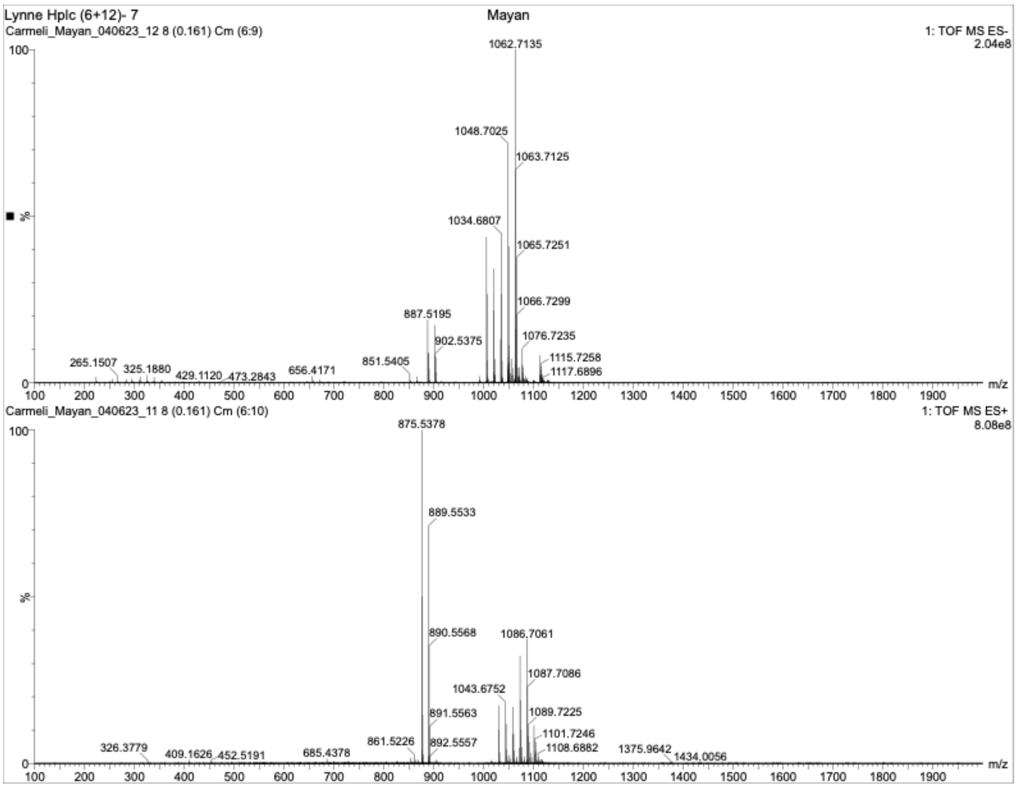

Figure S6. HRESIMS of fraction 8.

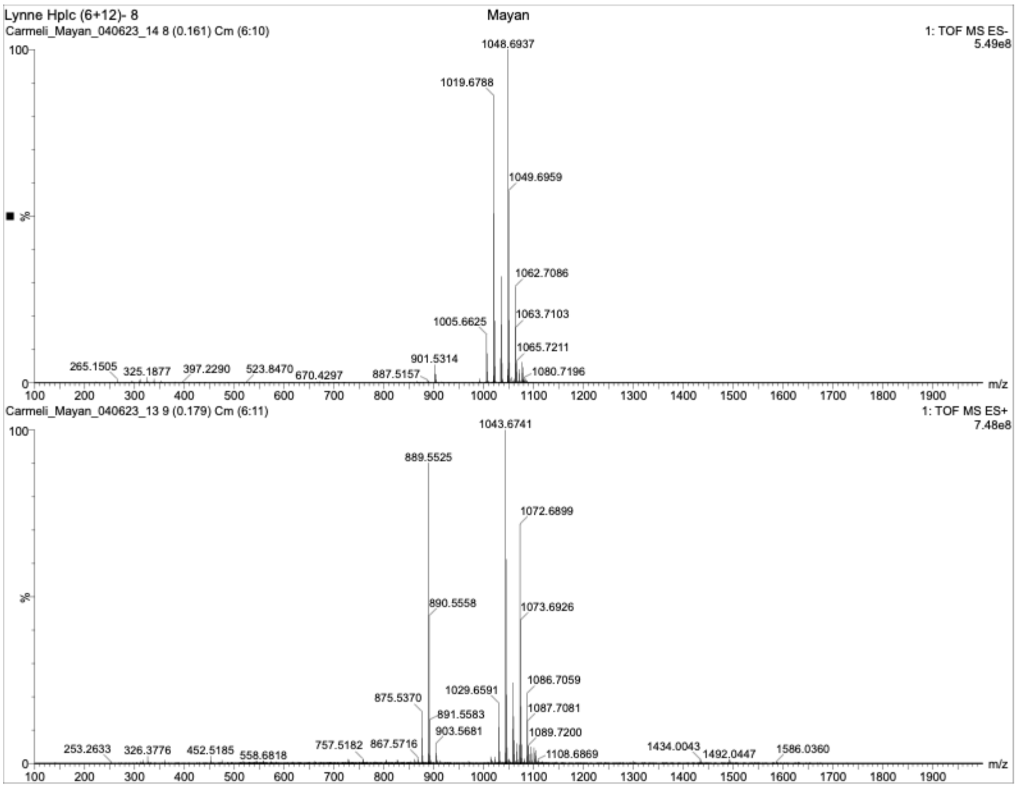

Figure S7. HRESIMS of fraction 9.

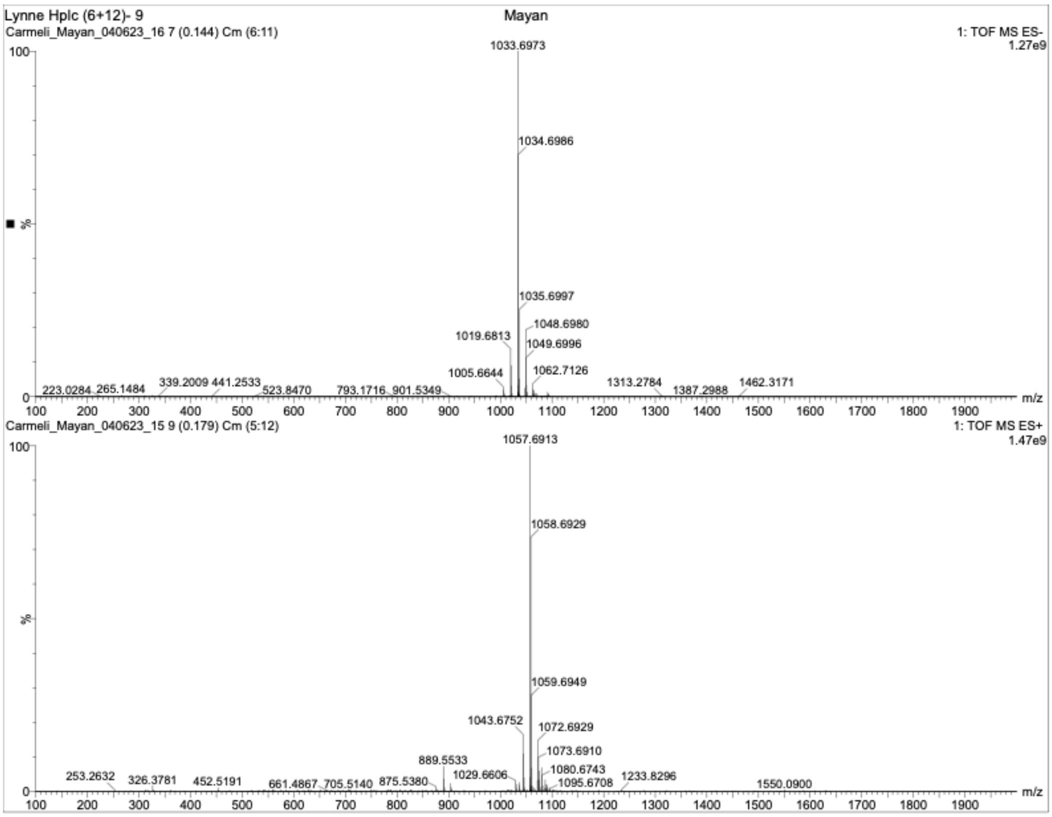

Figure S8. HRESIMS of fraction 10.

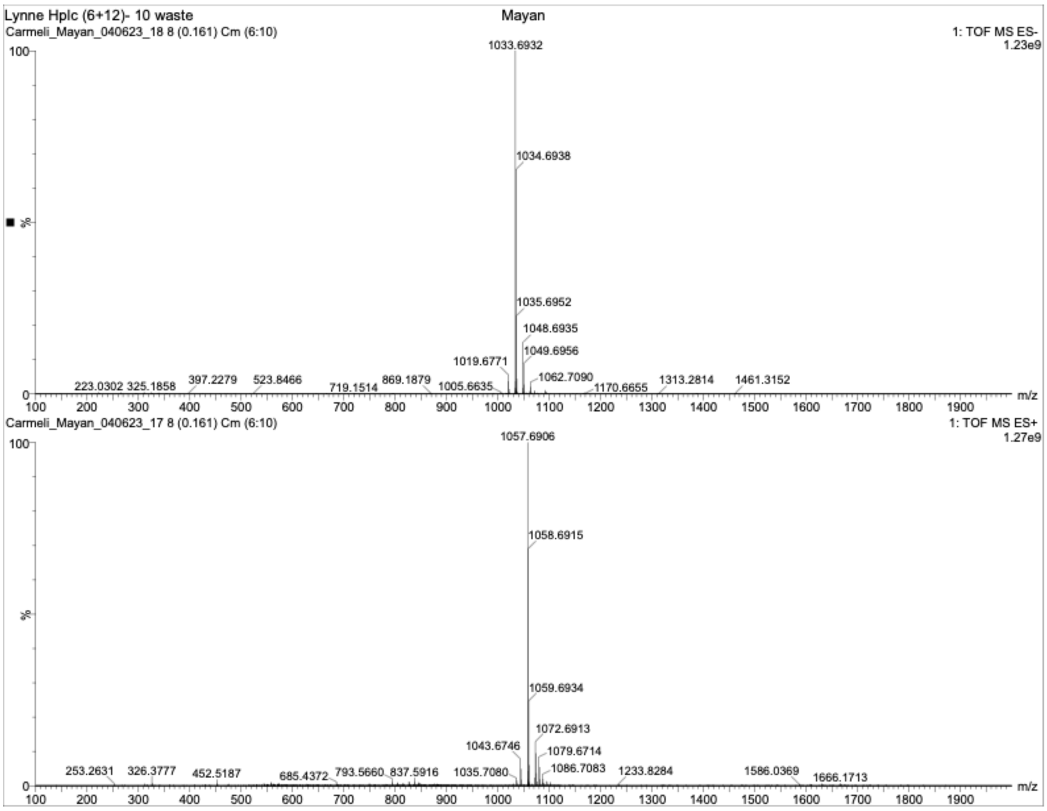

Figure S9. HRESIMS of fraction M-11-8.

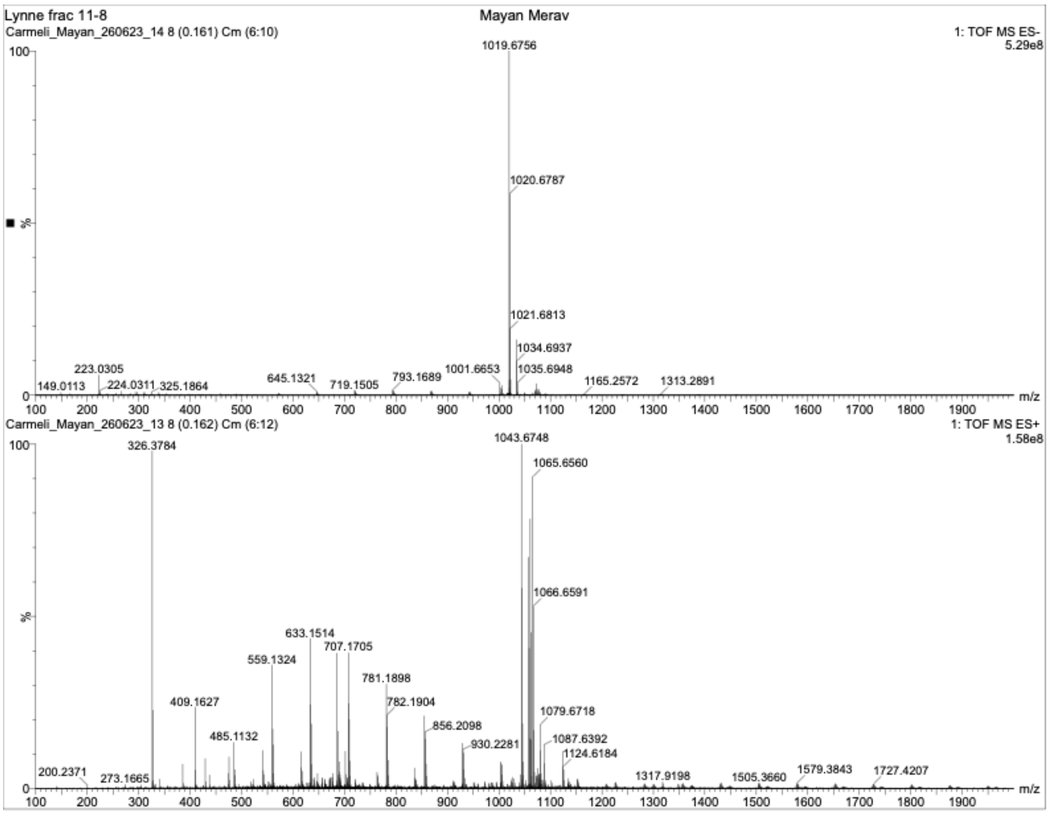

Figure S10. ^{1}H NMR (500 MHz) spectrum of fraction 9 in Pyr-d_5.

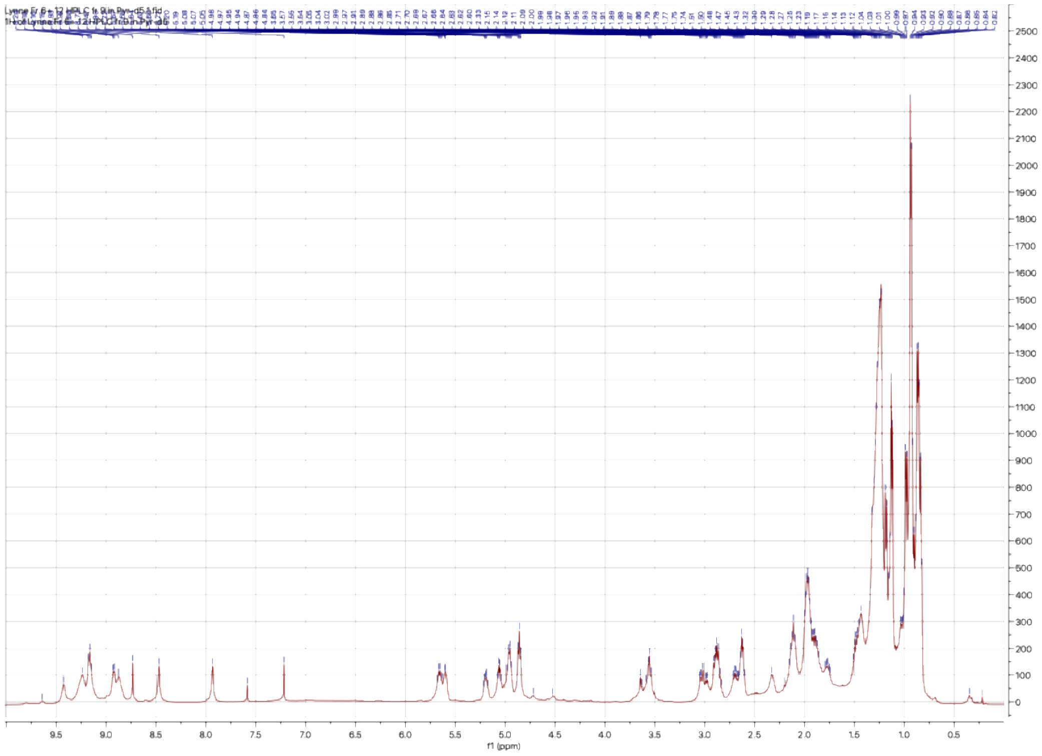

Figure S11. ^{13}C NMR (125 MHz) spectrum of fraction 9 in Pyr-d_5.

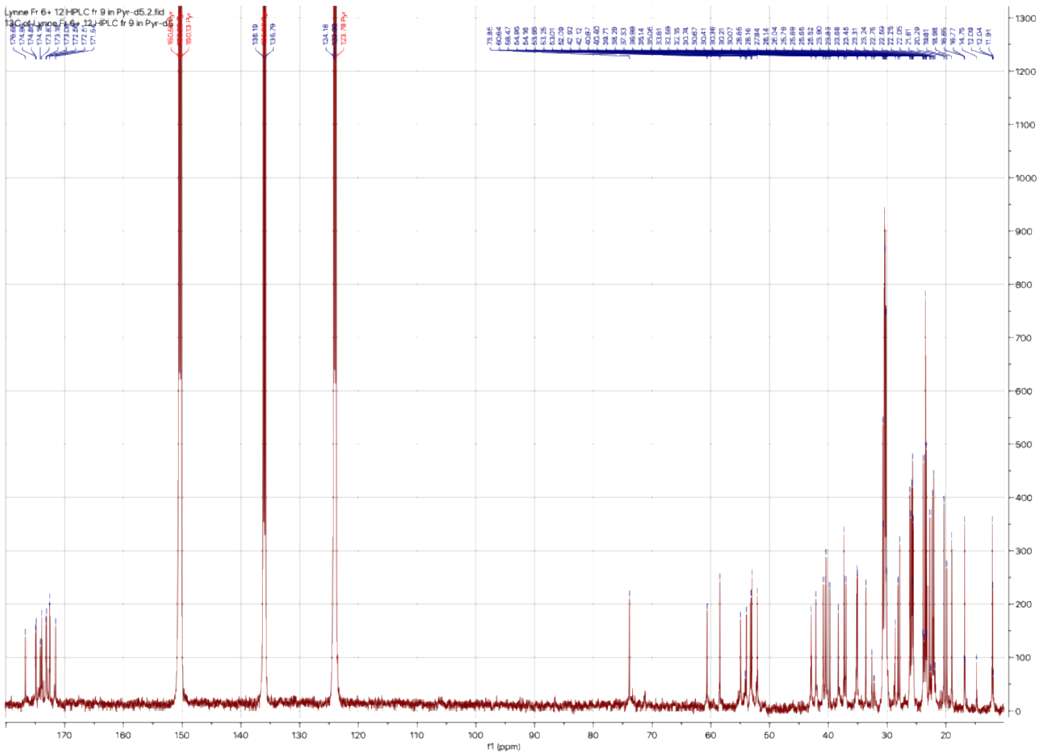

Figure S12. HSQC spectrum of fraction 9 in Pyr-d_5.

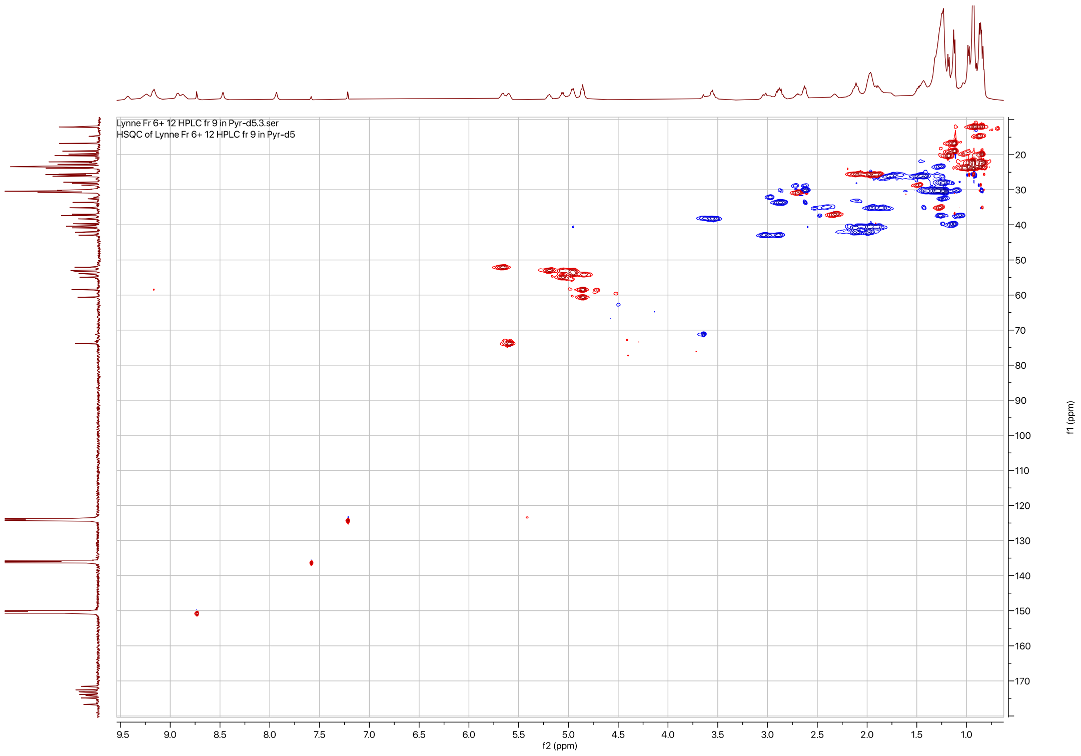

Figure S13. HMBC spectrum of fraction 9 in Pyr-d_5.

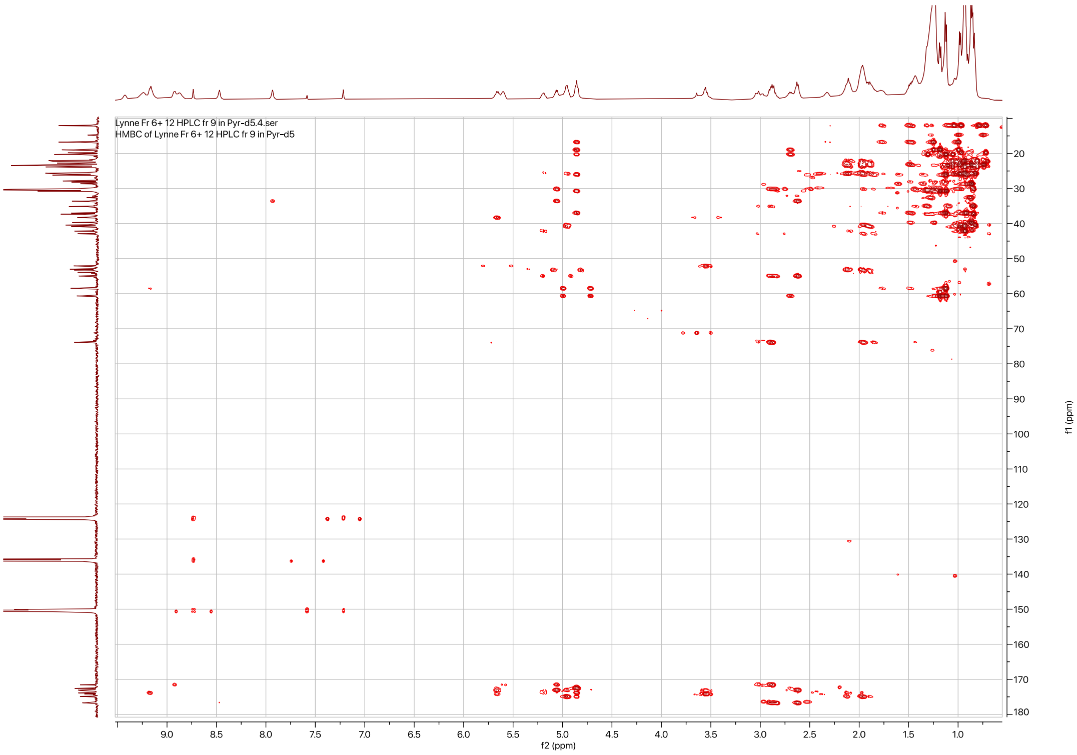

Figure S14. COSY spectrum of fraction 9 in Pyr-d_5.

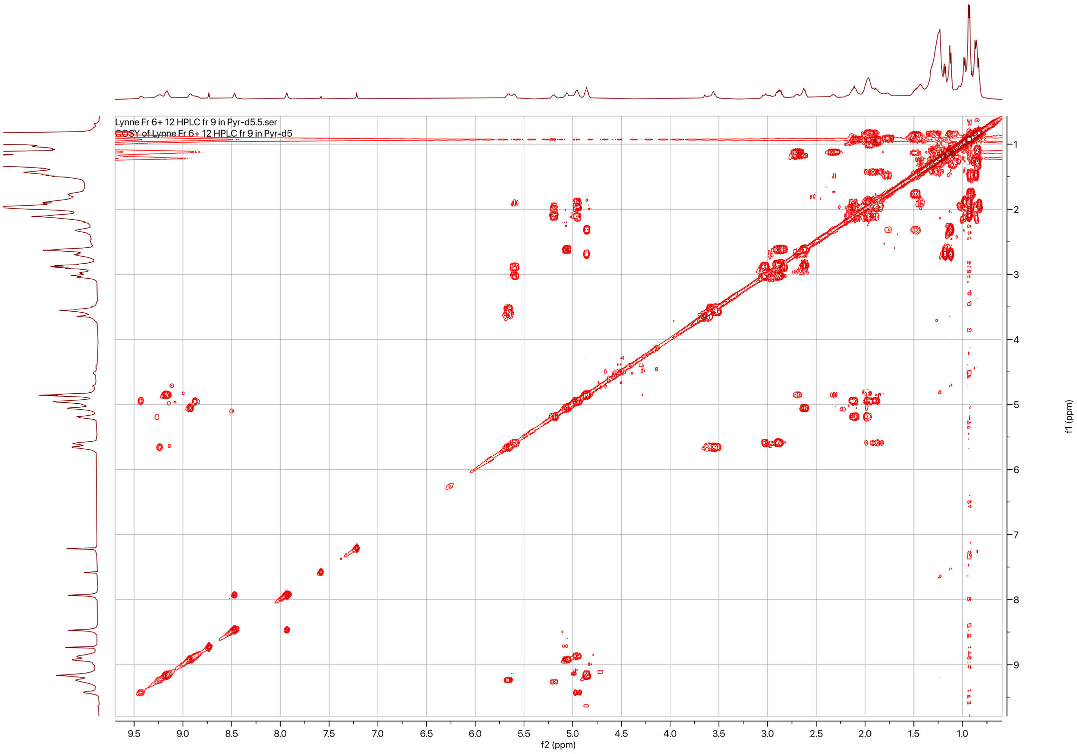

Figure S15. TOCSY spectrum of fraction 9 in Pyr-d_5.

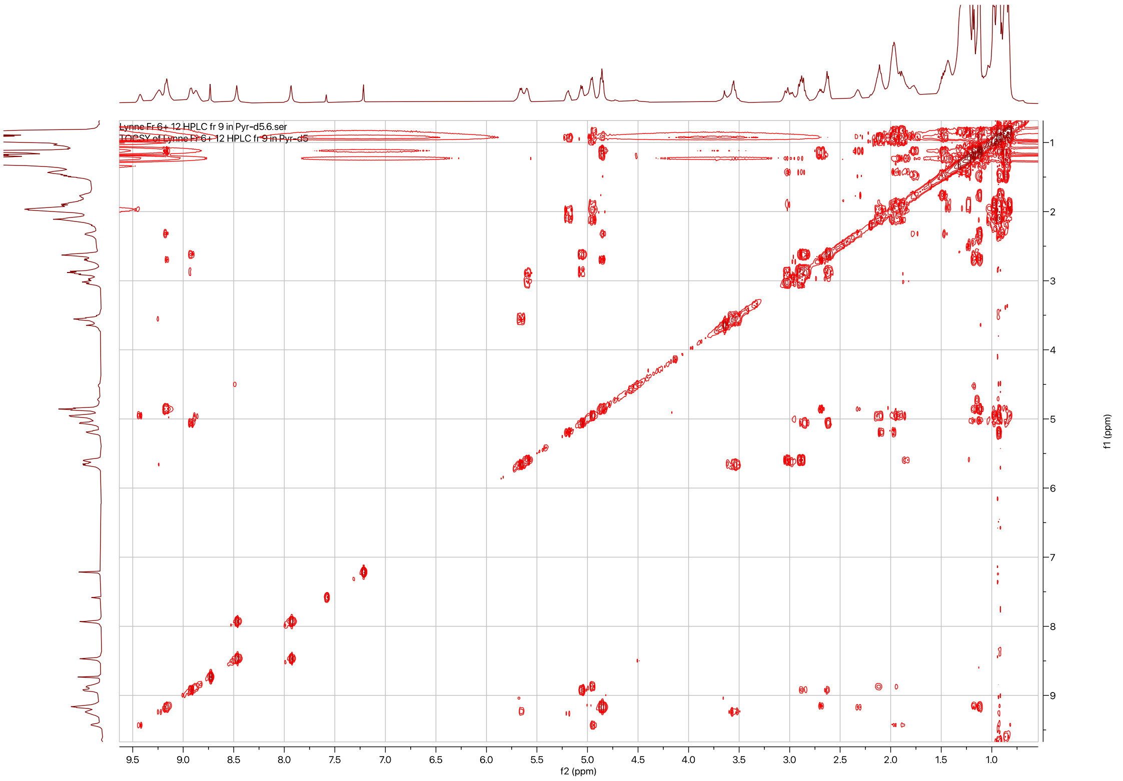

Figure S16. ROESY spectrum of fraction 9 in Pyr-d_5.

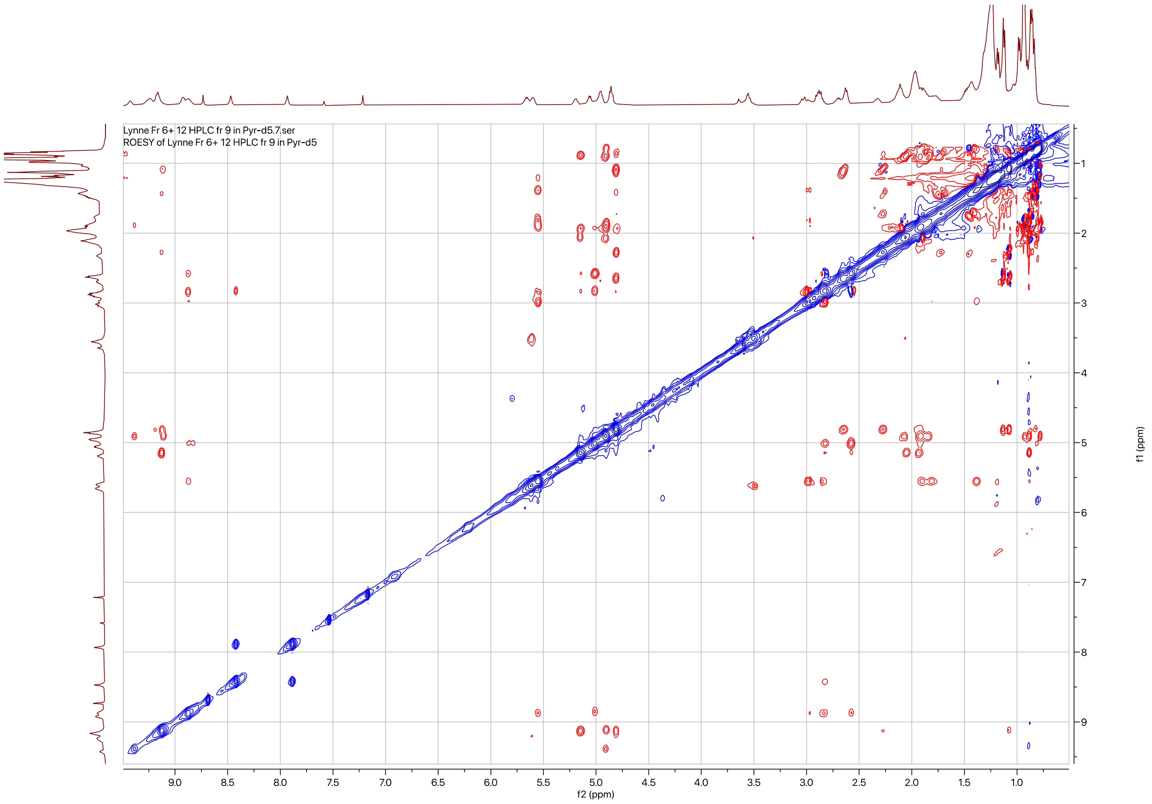

Figure S17. Structure and key HMBC correlations of the major component of fraction 9.

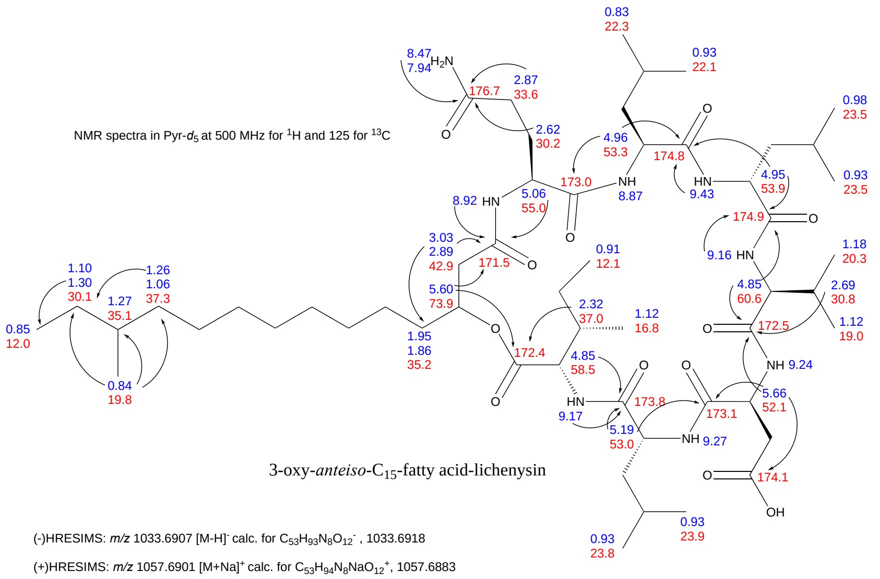

Table S1. NMR data of the major component of fraction B-6+M-12-HPLC-9 in Pyr-*d*5

Position	$\delta_C{}^a$	$\delta_H{}^b$	HMBC	COSY	TOCSY	ROESY
^{1}FA-1	171.5, C		^{1}FA-2a,2b,3, ^{2}Gln-2,2-NH			
^{1}FA-2a	42.9, CH_2	3.03, d	^{1}FA-3,4a, 4b	^{1}FA-2b,3	^{1}FA-2b,3, 4a,4b,5	^{1}FA-2b,4a, 4b,5
2b		2.89, m		^{1}FA-2a,3	^{1}FA-2a,3, 4a, 4b,5	^{1}FA-2a,4a, 4b,5
^{1}FA-3	73.9, CH	5.60, brm	^{1}FA-2a,2b, 4a,4b,5	^{1}FA-2a,2b, 4a,4b,	^{1}FA-2a,2b, 4a,4b,5	^{1}FA-2a,2b, 4a,4b,5,6
^{1}FA-4a	35.2, CH_2	1.95, m	^{1}FA-2a,2b, 5	^{1}FA-3,5		
4b		1.86, m		^{1}FA-3,5		
^{1}FA-5	30.4, CH_2	1.42, m				
^{1}FA-6	30.4, CH_2	1.31, m				

^{1}FA-7	30.4, CH$_2$	1.31, m				
^{1}FA-8	30.4, CH$_2$	1.25, m				
^{1}FA-9	30.4, CH$_2$	1.25, m				
^{1}FA-10	30.7, CH$_2$	1.21, m				
^{1}FA-11a<br>11b	37.3, CH$_2$	1.26, m<br>1.06, m	FA-12,13a, 13b,15			
^{1}FA-12	35.1, CH	1.27, m	^{1}FA-13a, 13b,15			
^{1}FA-13a<br>13b	30.1, CH$_2$	1.30, m<br>1.10, m	^{1}FA-11a, 12,15	^{1}FA-14<br>^{1}FA-14		
^{1}FA-14	12.0, CH$_3$	0.85, t	^{1}FA-13a, 13b	^{1}FA-13a, 13b		
^{1}FA-15	19.8, CH$_3$	0.84, d	^{1}FA-12, 13b	^{1}FA-13a		
^{2}Gln-1	173.0, C		^{2}Gln-2,4 ^{3}Leu-2			
^{2}Gln-2	55.0, CH	5.06, q	^{2}Gln-NH	^{2}Gln-NH,3	^{2}Gln-NH,3, 4	^{2}Gln-3,4, ^{4}Leu-3a,3b,
^{2}Gln-NH		8.92, d		^{2}Gln-2	^{2}Gln-2,3a,3b	^{1}FA-2a,2b, 3, ^{2}Gln-2,3, ^{5}Val-2,3
^{2}Gln-3	30.2, CH$_2$	2.62, m	^{2}Gln-2,4	^{2}Gln-2,NH	^{2}Gln-4	
^{2}Gln-4	33.6, CH$_2$	2.87, m	^{2}Gln-2,3,5-NHb	^{2}Gln-NH,3	^{2}Gln-3	
^{2}Gln-5	176.7, C		^{2}Gln-3,4,5-NH$_2$			
^{2}Gln-5-NH$_2$ a<br>b		8.47, s<br>7.94, s		^{2}Gln-5-NHb,<br>^{2}Gln-5-NHa	^{2}Gln-5-NHb,<br>^{2}Gln-3,4, 5-NHa	^{2}Gln-5-NHb*ex*,<br>^{2}Gln-3,4, 5-NHa*ex*
^{3}Leu-1	174.8, C		^{3}Leu-2,3a, 3b, ^{4}Leu-2			
^{3}Leu-2	53.2, CH	4.96, m	^{3}Leu-3a,3b, ^{3}Leu-6	^{3}Leu-NH,	^{3}Leu-NH, 3a,5	^{2}Gln-3,4, ^{4}Leu-3a,3b ^{3}Leu-5

^{3}Leu-NH		8.87, brs		^{3}Leu-2	^{3}Leu-2,3a, 6	^{2}Gln-2,3,4, ^{3}Leu-2,3a, 3b, ^{4}Leu-3a,3b
^{3}Leu-3a<br>3b	40.3, CH_2	1.96, m<br>1.89, m	^{3}Leu-2,4, 5,6	^{3}Leu-2,3b<br>^{3}Leu-2, 3a	^{3}Leu-2,3b<br>^{3}Leu-2,3a	^{3}Leu-NH, ^{4}Leu-2<br>^{3}Leu-NH, ^{4}Leu-2
^{3}Leu-4	25.6, CH	1.96, m	^{3}Leu-2,3a, 3b,5,6	^{3}Leu-2,5,6	^{3}Leu-2,3b, 5,6	^{4}Leu-NH, ^{3}Leu-5,6
^{3}Leu-5	22.3, CH_3	0.83, d		^{3}Leu-4		
^{3}Leu-6	22.1, CH_3	0.93, d		^{3}Leu-4		
^{4}Leu-1	174.9, C		^{5}Val-2,NH, ^{4}Leu-3a			
^{4}Leu-2	53.9, CH	4.95, m	^{4}Leu-3b,4, 5,6	^{4}Leu-NH, 3b,4	^{4}Leu-NH, 3b,4,5,6	^{4}Leu-3a, 3b,5,6
^{4}Leu-NH		9.43, brs		^{4}Leu-2	^{4}Leu-2,3b, 4,5,6	^{4}Leu-2,3b, 4
^{4}Leu-3a	40.8, CH_2	, m22.1	^{4}Leu-2,4,5, 6	^{4}Leu-2,3b, 4,5,6	^{4}Leu-2,3b, 4,5,6	^{3}Leu-NH, ^{4}Leu-5,6 ^{4}Leu-NH
3b		1.96, m		^{4}Leu-2,3a, 4	^{4}Leu-2,3a, 4,5,6	
^{4}Leu-4	25.7, CH	1.89, m	^{4}Leu-2,3a, 3b,5,6	^{4}Leu-3a,3b, 5,6	^{4}Leu-2,3a, 3b,5,6	
^{4}Leu-5	23.5, CH_3	0.98, d		^{4}Leu-4		
^{4}Leu-6	23.5, CH_2	0.93, d		^{4}Leu-4		
^{5}Val-1	172.5, C		^{5}Val-2,NH, 3			
^{5}Val-2	60.6, CH	4.85, t	^{5}Val-NH,3, 4,5	^{5}Val-NH,3	^{5}Val-NH,3, 4,5	^{1}FA-2a,2b, ^{5}Val-3,
^{5}Val-NH		9.16, d		^{5}Val-2	^{5}Val-2,3, 4,5	^{4}Leu-2, ^{5}Val-3,5
^{5}Val-3	30.8, CH	2.69, m	^{5}Val-2,4,5	^{5}Val-2,4,5	^{5}Val-2,4,5	
^{5}Val-4	20.3, CH_3	1.18, d	^{5}Val-2,4,5	^{5}Val-3		

^{5}Val-5	19.0, CH$_3$	1.12, d	^{5}Val-2,3,4	^{5}Val-3		
^{6}Asp-1	173.1, C		^{6}Asp-2,3, ^{7}Leu-2			
^{6}Asp-2	52.1, CH	5.66, q	^{6}Asp-3	^{6}Asp-NH,3	^{6}Asp-NH,3	^{6}Asp-3, ^{1}FA-2a,2b
^{6}Asp-NH		9.24, brm		^{6}Asp-2	^{6}Asp-2,3	^{5}Val-2,3, ^{6}Asp-2,3, ^{7}Leu-3a
^{6}Asp-3	38.3, CH$_2$	3.55, m	^{6}Asp-2	^{6}Asp-2		
^{6}Asp-4	174.1, C		^{6}Asp-2,3			
^{7}Leu-1	173.8, C		^{8}Ile-2,NH, ^{7}Leu-2,3a, 3b			
^{7}Leu-2	53.0, CH	5.19, m	^{7}Leu-5	^{7}Leu-NH, 3a,3b	^{7}Leu-NH, 3a,3b,4,5	^{1}FA-2b, ^{7}Leu-3a,3b, 4,5,6
^{7}Leu-NH		9.27, m		^{7}Leu-2,	^{7}Leu-2,3a, 3b,4,5	^{6}Asp-3, ^{7}Leu-3a
^{7}Leu-3a<br>3b	42.1, CH$_2$	2.11, m<br>1.98, m	^{7}Leu-2,45, 6	^{7}Leu-2,3b<br>^{7}Leu-2,3a		
^{7}Leu-4	25.4, CH	, m02.1	^{7}Leu-2,5,6	^{7}Leu-5,6	^{7}Leu-3b,5, 6	
^{7}Leu-5	23.9, CH$_3$	0.93, d		^{7}Leu-4		
^{7}Leu-6	23.8 CH$_3$	0.93, d		^{7}Leu-4		
^{8}Ile-1	172.4, C		^{8}Ile-NH,3, ^{1}FA-3			
^{8}Ile-2	58.5, CH	4.85, t	^{8}Ile-NH,3, 4a,4b,5	^{8}Ile-NH,3	^{8}Ile-NH,3,5	^{1}FA-2a,2b, ^{8}Ile-3,4a, 4b,5,6
^{8}Ile-NH		9.17, d		^{8}Ile-2	^{8}Ile-2,3,5	^{7}Leu-2, ^{8}Ile-2,3,4a, 4b, ^{1}FA-2b
^{8}Ile-3	37.0, CH	2.32, m	^{8}Ile-2,4b,5, 6	^{8}Ile-2,5	^{8}Ile-2, 4a, 4b,5,6,	
^{8}Ile-4a<br>4b	26.0, CH$_2$	1.77, m<br>1.48, m	^{8}Ile-2,5,6	^{8}Ile-3,4b,6<br>^{8}Ile-3,4a,6		

[8]Ile-5	16.8, CH_3	1.12, d	[8]Ile-2,3,4a, 4b	[8]Ile-3	
[8]Ile-6	12.1, CH_3	0.91, t	[8]Ile-3,4a,4b	[8]Ile-3,4a,4b	[8]Ile-3,4a,4b

[a]125 MHz for ^{13}C; [b]500 MHz for ^{1}H.

Table S2. Different medium compositions used to cultivate *C. nicaeensis*-associated bacteria.

Medium	Composition
SCA	10 g L^{-1} soluble starch, 1 g L^{-1} casein [dissolved in 10 mL 1 M NaOH and neutralized after addition by 10 mL 1 M HCl], 0.5 g L^{-1} K2HPO4, 20 g L^{-1} NaCl, 20 g L^{-1} agar
MA	Yeast extract 5g L^{-1}, Agar 20 g L^{-1}, peptone 5 g L^{-1}
ISP2	Yeast extract 4 g L^{-1}, malt extract 10 g L^{-1}, dextrose 4 g L^{-1}, Agar 20 g L^{-1}
LB	20 g L^{-1} LB broth, 18 g L^{-1} agar

Table S3. PCR reaction mixture and cycling parameters of 16S amplification of *C. nicaeensis* associated bacteria.

PCR mix

PCR mix (16S)	Concentration	Volume per sample
Dreamtaq master mix (Thermofisher©)		25 µl
primers (F/R)	10 mM	5 µl
UPW		13 µl
DNA		2 µl
Total volume		45 µl

PCR program

Step	Temperature	Time [min]	Notes
1	95°C	3	Initial heating
2	95°C	0.5	Denaturation
3	59.5°C	0.5	Annealing
4	72°C	1	Extension, repeat stages 2-4, 26 rounds
5	72°C	10	
6	10°C	Until samples were taken out	
